# Organizational readiness for change: A systematic review of the healthcare literature

**DOI:** 10.1177/26334895251334536

**Published:** 2025-05-15

**Authors:** Laura Caci, Emanuela Nyantakyi, Kathrin Blum, Ashlesha Sonpar, Marie-Therese Schultes, Bianca Albers, Lauren Clack

**Affiliations:** 1 Institute for Implementation Science in Health Care, Medical Faculty, 27217 University of Zurich, Zurich, Switzerland; 2 Department of Infectious Diseases and Hospital Epidemiology, University Hospital of Zurich, Zurich, Switzerland

**Keywords:** organizational readiness for change, organizational climate, implementation theories, implementation science, health care, systematic review

## Abstract

**Background:**

Organizational readiness for change (ORC), referring to psychological and behavioral preparedness of organizational members for implementation, is often cited in healthcare implementation research. However, evidence about whether and under which conditions ORC is relevant for positive implementation results remains ambiguous, with past studies building on various theories and assessing ORC with different measures. To strengthen the ORC knowledge base, we therefore identified factors investigated in the empirical literature alongside ORC, or as mediators and/or moderators of ORC and implementation.

**Method:**

We conducted a systematic review of experimental, observational, and hybrid studies in physical, mental, and public health care that included a quantitative assessment of ORC and at least one other factor (e.g., ORC correlate, predictor, moderator, or mediator). Studies were identified searching five online databases and bibliographies of included studies, employing dual abstract and full text screening. The study synthesis was guided by the Consolidated Framework for Implementation Research integrated with the Theory of ORC. Study quality was appraised using the Mixed Methods Appraisal Tool.

**Results:**

Of 2,907 identified studies, 47 met inclusion criteria, investigating a broad range of factors alongside ORC, particularly contextual factors related to individuals and the innovation. Various ORC measures, both home-grown or theory-informed, were used, confirming a lack of conceptual clarity surrounding ORC. In most studies, ORC was measured only once.

**Conclusions:**

This systematic review highlights the broad range of factors investigated in relation to ORC, suggesting that such investigation may enhance interpretation of implementation results. However, the observed diversity in ORC conceptualization and measurement supports previous calls for clearer conceptual definitions of ORC. Future efforts should integrate team-level perspectives, recognizing ORC as both an individual and team attribute. Prioritizing the use of rigorous, repeated ORC measures in longitudinal implementation research is essential for advancing the collective ORC knowledge base.

## Plain Language Summary

***A systematic review of studies investigating healthcare organizations' members' preparedness for implementing change into routine health care***. Organizational readiness for change (ORC) describes whether members of an organization, for example, hospital doctors or nurses, feel prepared for implementing a change, for example using a new therapy or clinical procedure, into daily work routines. Typically, it is assumed that when ORC is high, staff are on board with a change, and meet expectations to make the implementation happen. In this way, ORC is often used to explain why an implementation was successful—or not. However, researchers often define and measure ORC in different ways. This makes it difficult to determine whether, and under what conditions ORC influences implementation. We therefore conducted a systematic literature review to identify factors that have been investigated in combination with ORC. Our aim was to learn how these factors may help to better understand ORC's role in implementing interventions in health care. We identified 47 relevant studies. Across these, many different factors were examined in combination with ORC. Multiple factors related to individuals involved in implementation (e.g., staff attitudes), and to the implemented innovation (e.g., its acceptability). As these factors were diverse, it is still unclear how relevant ORC is for implementation in health care. Moreover, it is important that researchers explain clearly how they define ORC, what theories they use, and how they measure ORC. We found that ORC was typically measured once, before implementation, confirming limitations of earlier ORC studies. This systematic literature review points to ORC as a relevant concept when examining implementation in health care. However, in doing so, it is important to not only study individual factors, but group characteristics, as implementation is a team sport. Researchers and implementers should also consider ORC over the long term and measure it rigorously. This will help to better understand the role of ORC in implementing effective interventions in health care.

## Introduction

Organizational readiness for change (ORC) is a concept frequently considered in implementation science—often described as a linchpin between intentions, actual engagement, and observed outcomes in the implementation of new practices. When explaining the results of an implementation, be they positive, negative, or null findings, implementation scholars often point to ORC as one potential reason for either of these scenarios (Armenakis et al., 1993; Weiner, 2020). As a multidimensional concept, ORC is defined as “organizational members’ psychological and behavioral preparedness to implement change” (Weiner, 2020, p. 217). In aiming to explain that only half of all evidence-supported interventions (ESIs) make it into routine healthcare practice, scholars have suggested ORC but also contextual factors (Bauer & Kirchner, 2020), such as teamwork (Taylor et al., 2011), leadership (Aarons et al., 2016; Reichenpfader et al., 2015; Taylor et al., 2011), perceptions of trust (Vakola, 2013), implementation climate (Williams et al., 2020), resource availability (Helfrich et al., 2007), structural organizational characteristics (Taylor et al., 2011), or organizational climate (Kelly et al., 2018) as potential contributing factors. A deeper examination of these factors can help to accelerate and enhance ESI implementation in health care. This applies particularly to ORC, as claims about its importance often have not been based on the use of nuanced theories or empirical prospective designs clearly linking ORC to implementation outcomes (Scaccia et al., 2015; Weiner, 2020).

Current evidence on how ORC influences ESI implementation in health care is ambiguous (Weiner, 2020). For example, Noe et al. (2014) used the *Organizational Readiness to Change Assessment* (ORCA; Helfrich et al. (2009)) to assess ORC and capacity to provide culturally competent services for American Indian and Alaska Native veterans in Veterans Affairs facilities. The authors found that no ORCA subscale predicted implementation of native-specific services. Contrarily, Becan et al. (2012) reported a positive association among subscales of the *ORC Scale* (Lehman et al., 2002) and ESI adoption in substance use treatment settings. Possible explanations for why these two studies reach different conclusions about the role of ORC are manyfold.

First, the concept of ORC remains fuzzy despite many attempts to define its core and essence. In general, ORC conceptualizations entail psychological components (e.g., motivation), structural components (e.g., resources), or both. In Weiner's psychologically framed *Theory of ORC* (*TORC*), ORC consists of change commitment and change efficacy, with *change commitment* being organizational members’ shared resolve to show behaviors required by the change and *change efficacy* describing their shared sense of capability to pursue change behaviors (Weiner, 2009). According to the *TORC*, higher ORC scores result in higher change-related effort among organizational members, leading to positive implementation results (Weiner, 2009, 2020). However, this is only one of many definitions of ORC. Scaccia et al. (2015) define ORC as “the extent to which an organization is both willing and able to implement a particular innovation” (p. 485). Armenakis et al. (1993) define ORC as “organizational members’ beliefs, attitudes, and intentions regarding the extent to which changes are needed and the organization's capacity to successfully make those changes” (p. 681).

Second, based on different ORC conceptualizations, different ORC measurement tools have been developed. In a systematic review, Miake-Lye et al. (2020) identified 29 measures used for ORC assessments in health care. These measures range in scope and were developed for different settings. For instance, the 116-item *ORC Scale* was developed in an addiction treatment setting (Lehman et al., 2002), whereas the 74-item *ORCA* (Helfrich et al., 2009) was developed within three quality improvement projects in the U.S. Veterans Health Administration, while the 41-item *Readiness for Organizational Change Scale* was developed in a government organization setting (Holt, Armenakis, Feild, & Harris, 2007). More recently, Shea et al. (2014) developed the pragmatic 12-item *Organizational Readiness for Implementing Change* (ORIC) scale across different settings.

Third, differing study needs and contexts have led to a substantial number of homegrown or adapted, often single-use ORC measures (Miake-Lye et al., 2020), contributing to a landscape of measures with limited validity and reliability (Gagnon et al., 2014; Holt, Armenakis, Harris, & Feild, 2007; Weiner et al., 2008). In a more recent review, Weiner et al. (2020) state that 72% of ORC measures were used once only.

Finally, scholars continue to measure ORC either retrospectively or at baseline only, without prospectively linking ORC to implementation outcomes. This phenomenon is likely perpetuated by misconceptions of “readiness” as a static, binary condition (i.e., present or absent) to be assessed prior to implementation as a precondition for pursuing efforts. This is problematic, as ORC fluctuates with the ever-changing circumstances of healthcare settings and therefore merits longitudinal measurement (Scaccia et al., 2015). Retrospective ORC measurements may therefore misrepresent the reality at the time of change initiation. Baseline ORC measurements come with a similar limitation, as baseline circumstances may fail to reflect the conditions present during implementation activities or when implementation outcomes are measured. These shortcomings hamper the interpretation of studies about the role of ORC for implementation (Weiner et al., 2020). Consequently, the importance of ORC for implementation in health care remains unclear.

These central challenges become apparent in the two ORC studies introduced above. Noe et al. (2014) and Becan et al. (2012) were based on different theories, which led to the use of different ORC measures. As summarized in Table 1, Noe et al. (2014) used the ORCA (Helfrich et al., 2009), developed based on the Promoting Action on Research in Health Services model (Kitson et al., 1998; Rycroft-Malone, 2004), which is reflected in the ORCA scales: *evidence*, *context*, and *facilitation*. Becan et al. (2012) measured ORC with the ORC scale (Lehman et al., 2002). Its underlying model is the Program Change Model (Simpson, 2002), which contains four domains: *motivation for change, adequacy of resources, staff attributes,* and *organizational climate.*

**Table 1:** Illustration of central challenges with two exemplary studies using different measures for organizational readiness for change

	Noe et al. (2014)	Becan et al. (2012)
Measure used	Organizational Readiness to Change Assessment (Helfrich et al., 2009)	Organizational Readiness for Change Scale (Lehman et al., 2002)
Measure scales or domains	Evidence, context, and facilitation	Motivation for change, adequacy of resources, staff attributes, and organizational climate
Underlying theory, model, or framework	Promoting Action on Research in Health Services (Kitson et al., 1998; Rycroft-Malone, 2004)	Program Change Model (Simpson, 2002)

Taken together, the evidence surrounding ORC is ambiguous, offering limited knowledge about whether, how, and under which conditions ORC influences implementation. Moreover, other factors may influence implementation or moderate or mediate the role of ORC for implementation in health care. Furthermore, the importance of ORC can vary based on the degree to which implementation requires behavior change, implementer or end-user familiarity with these behaviors, or the use of implementation strategies.

Against this background, the overarching aim of this systematic literature review (SLR) is to identify studies reporting factors that—in relation to ORC—are potentially important for implementing change in healthcare settings. This SLR is part of a larger project aimed at identifying whether and under which conditions ORC influences change-related effort in implementing infection prevention and control practices in acute care. Its results will be used for a Coincidence Analysis, a case-based method rooted in Boolean algebra, designed to find factors that are necessary and sufficient for a given outcome (Whitaker et al., 2020). The SLR is therefore focused on identifying the breadth of factors—in relation to ORC—potentially influencing change-related effort as defined in the TORC (Weiner, 2020), further described below.

The research questions (RQs) to be answered are:

1. *What factors have been investigated in the empirical literature in relation to ORC?*
2. *What factors have been investigated in the empirical literature as possible mediators or moderators of the relationship between ORC and implementation?*

## Methodology

We conducted an SLR allowing for the inclusion of experimental, nonexperimental, and quasi-experimental studies. This SLR was preregistered on PROSPERO (CRD42023368072) and reported based on the Preferred Reporting Items for Systematic Reviews and Meta-Analyses (PRISMA) guidelines (Page et al., 2021). The PRISMA checklist is included in Supplemental Material A.

### Theoretical underpinnings

**Figure 1:**
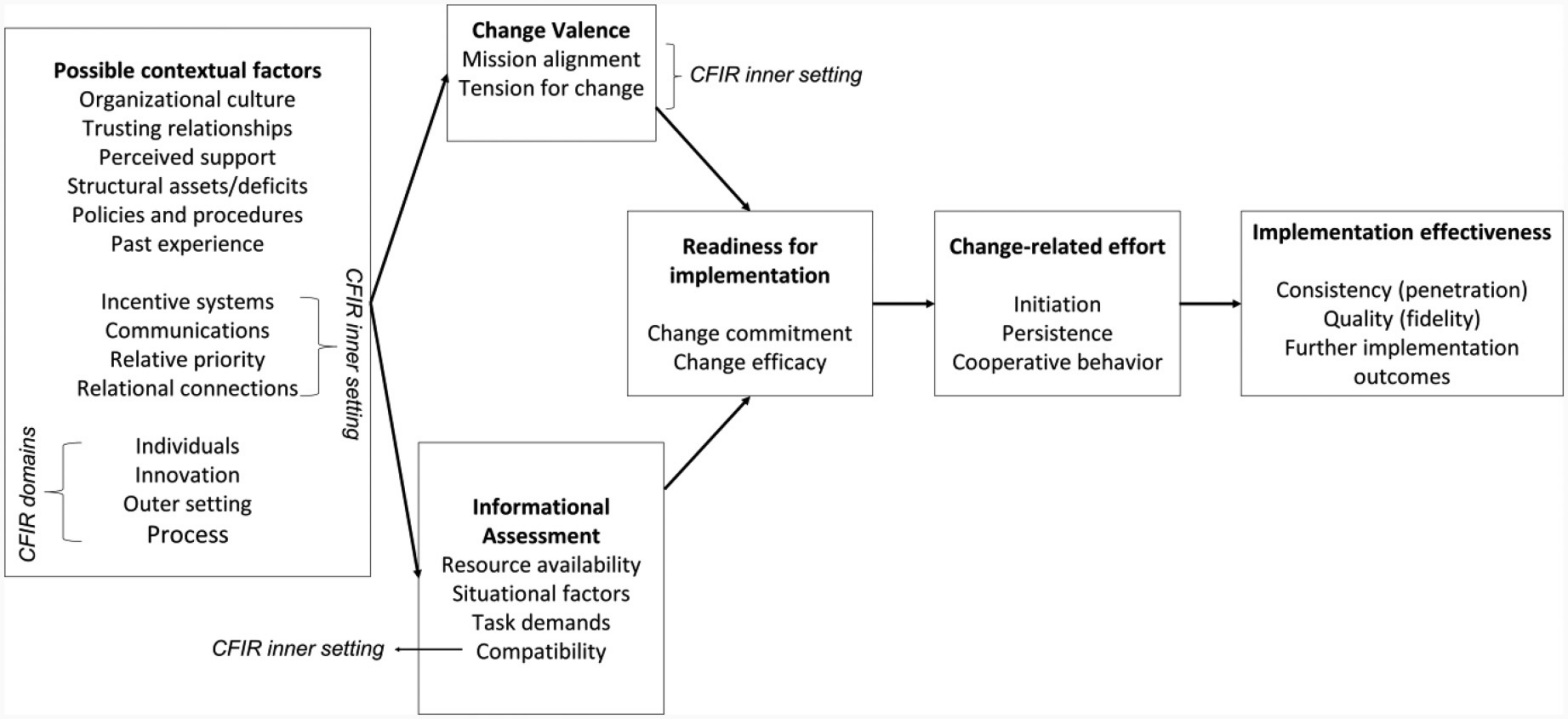
Guiding TORC-CFIR integrated framework used for this systematic review (adapted from Damschroder et al., 2022; Weiner, 2020). Further implementation effectiveness outcomes can be added to the TORC-CFIR framework as needed (e.g., acceptability, appropriateness, feasibility; Proctor et al., 2011)

*Note.* TORC = Theory of Organizational Readiness for Change; CFIR = Consolidated Framework for Implementation Research.

An integrated framework, including the TORC (Weiner, 2020) and the updated *Consolidated Framework for Implementation Research* (CFIR; Damschroder et al., 2022) guided the synthesis of included studies (Figure 1).

The TORC describes *change efficacy* and *change commitment* as the two main ORC components. ORC is promoted by *change valence* and *informational assessment,* which are theorized as direct ORC determinants. *Change valence* describes the value that organizational members assign to the change, and *informational assessment* describes their cognitive combination of information about task demands, resource availability, and situational factors. Their interplay is assumed to be influenced by *possible contextual factors* that influence ORC indirectly through *change valence* and *informational assessment*. *Possible* c*ontextual factors* within the TORC include organizational culture and climate, policies and procedures, perceived organizational support, trusting relationships, and past experience. For example, depending on whether the change aligns with cultural values or not, organizational culture could influence change valence and affect ORC (Weiner, 2020).

The usability of the TORC as guiding theory was enhanced by integrating CFIR domains. The CFIR is a determinant framework that outlines potential implementation barriers and facilitators. To combine the TORC and the CFIR, the CFIR domains *individuals*, *innovation*, *outer setting,* and *implementation process* were integrated into the *possible contextual factors* of the TORC. In anticipation of inner setting determinants playing a central role in ORC, we also integrated constructs of the CFIR *inner setting* domain into the TORC elements *possible contextual factors*, *change valence,* and *informational assessment*. Integrating CFIR domains in this way allowed for specifying and broadening the range of potential factors relevant to the RQs that guide this SLR. When TORC and CFIR terminologies overlapped, the term representing greater specificity regarding ORC and simultaneously minimizing ambiguity among factors was used (Table 2).

**Table 2:** Integration of TORC and CFIR inner setting terminology

TORC terminology	CFIR inner setting terminology	TORC-CFIR combination
Structural assets/deficits	Structural characteristics	Structural assets/deficits
Organizational culture	Culture	Organizational culture
Resource availability	Available resources Access to knowledge and information	Resource availability

*Note*. TORC = Theory of Organizational Readiness for Change; CFIR = Consolidated Framework for Implementation Research.

### Eligibility criteria

Studies eligible for inclusion had to be conducted in health care settings. Furthermore, a quantitative ORC measurement, and at least one additional reported factor (RF) had to be quantitatively or qualitatively studied in relation to ORC. Regardless of the emerging results of included studies, the investigation of RFs in relation to ORC could occur in two ways: RFs could be investigated as direct or indirect predictors or correlates of ORC or they could be examined as possible mediators or moderators of the relationship between ORC and implementation in included studies. Table 3 details eligibility criteria.

**Table 3:** Eligibility criteria for inclusion of full-text articles

	Included	Excluded
Phenomenon of interest—the concept of Organizational Readiness for Change (ORC)	Studies reporting on ORC related to the implementation of distinct practices, policies, or programs.	Studies reporting on ORC related to organizational change processes of structural nature, such as restructuring leadership, merging multiple organizations, or joining an association of organizations.
Phenomenon of interest—ORC assessment^1^	Studies measuring ORC quantitatively, at the level of the organization.	Studies solely investigating individual readiness for a change process, studies investigating ORC qualitatively, studies assessing ORC in technological terms, studies not using any ORC assessment.
Phenomenon of interest—RFs in relation to ORC	Studies that report investigating other factors in relation to ORC, be it as predictors or correlates of ORC, for example, a study investigating the association between ORC and organizational culture.	Studies reporting on ORC only.
Study setting	Studies conducted with healthcare workers in healthcare organizations operating in physical, mental, and public health settings.	Healthcare studies conducted in organizations operating in other settings, for example, schools, social care, industry, or churches.
Study design	All study designs (i.e., experimental, nonexperimental/observational, hybrid designs, etc.).	
Study methodology^1^	Quantitative or mixed-methods studies that include a quantitative ORC assessment.	Purely qualitative studies and mixed-methods studies that assessed ORC using qualitative methods only.
Publication type	Primary study reports.	Any other type of publication, for example, dissertations, book chapters, conference abstracts, systematic reviews, study protocols.
Publication date	All publication dates (up to August 2022).	
Study geographies	Studies conducted in all geographies.	
Study languages	Studies published in Danish, English, French, German, Italian, Norwegian, Spanish, or Swedish.	Studies published in other languages.

*Note.* RF = Reported Factors; PRISMA = Preferred Reporting Items for Systematic Reviews and Meta-Analyses.

**Figure 2:**
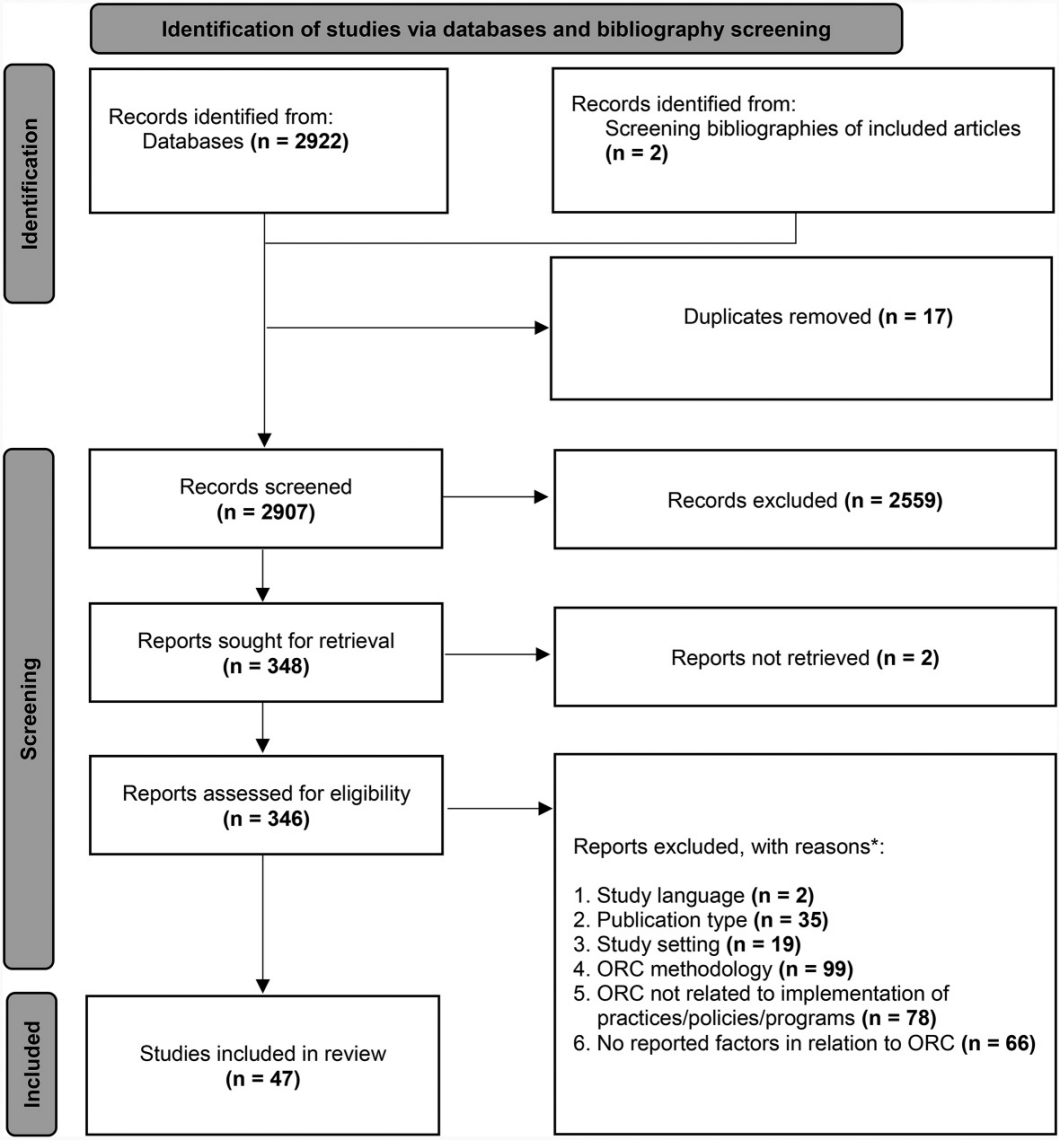
PRISMA flowchart. * Hierarchy for exclusion from top to bottom

*Note*. PRISMA = Preferred Reporting Items for Systematic Reviews and Meta-Analyses.

1 Both criteria match the same reason for full text exclusion in PRISMA flowchart (Figure 2).

### Information sources

A literature search was performed in August 2022. Five online databases were searched, displayed in Supplemental Material B. Additionally, two reviewers checked the bibliographies of included studies for further relevant articles.

### Search strategy

Key terms used in the search strategy were related to the following concepts and adapted for each database: healthcare, organization, readiness, change. Table 4 shows the search strategy used in MEDLINE. Supplemental Material B contains the search strategies used for each database.

**Table 4:** Search strategy for MEDLINE

Number	Query
S3	S1 AND S2
S2	(MH “Health Facilities+”) OR TI (health OR hospital* OR healthcare OR “primary care” OR “medical care”) OR AB (health OR hospital* OR healthcare OR “primary care” OR “medical care”) OR SO (health OR hospital* OR healthcare OR care)
S1	((MH “Organizational Innovation” OR TI ((organization* OR organisation* OR institution*) N6 (innovation* OR change OR intervention* OR transformation*)) OR AB ((organization* OR organisation* OR institution*) N6 (innovation* OR change OR intervention* OR transformation*))) AND (TI (readiness OR ready OR willing* OR preparedness) OR AB (readiness OR ready OR willing* OR preparedness)) OR ((TI ((organization* OR organisation* OR institution*) N6 (readiness OR ready OR willing* OR preparedness)) OR AB ((organization* OR organisation* OR institution*) N6 (readiness OR ready OR willing* OR preparedness))) AND (TI (innovation* OR change OR intervention* OR transformation*) OR AB (innovation* OR change OR intervention* OR transformation*))

### Selection process

Two reviewers independently screened titles, abstracts, and full texts in duplicate against predefined eligibility criteria (Table 3) using Covidence (Covidence systematic review software, 2022). Reasons for full-text exclusion were indicated. Disagreements were either solved by consensus discussion or by a third reviewer (B.A.).

### Data extraction

Pairs of independent reviewers extracted data from included studies using a predefined Microsoft Excel form. One reviewer (L.Ca.) extracted two additional studies identified through bibliography checks of included studies. Discrepancies were solved by consensus discussion. Data were extracted for: publication author and year, study setting, study methodology, study design, data collection, change implemented, and ORC measure used. Furthermore, we extracted information from methods and results sections of included studies on RFs investigated in relation to ORC (i.e., as predictors or correlates of ORC, or mediators or moderators of the relationship between ORC and implementation) and therefore relevant to our RQs.

### Study risk of bias assessment

Due to the variety of study designs eligible for this SLR, reviewer pairs independently appraised study quality using the Mixed Methods Appraisal Tool (MMAT) version 2018 (Hong et al., 2018), developed for use in qualitative studies, randomized controlled trials, nonrandomized studies, quantitative descriptive studies, and mixed-methods studies. Reviewers discussed disagreements until consensus was reached. The MMAT provides differing sets of five quality criteria for each study category, with criteria being rated with “Yes” when a criterion is met, “No” when a criterion is not met, and “Can’t tell” when there is insufficient information to judge whether a criterion is met. The authors of the MMAT discourage computing a numerical overall score of study ratings, as this would not provide information on specific low-quality aspects of the studies (Hong et al., 2018). Therefore, the MMAT was not used to inform in- or exclusion, or synthesis, but to provide indications of the overarching quality of included studies and their evidence, thereby helping to contextualize our findings in the broader landscape of ORC literature.

### Synthesis of findings

Two reviewers (B.A. and L.Ca.) conducted the synthesis of RFs in two steps:

1. Clustering of similar RFs: RFs were listed by name as reported in studies, and then grouped according to topical similarity, using construct definitions provided in the CFIR (Damschroder et al., 2022) and the TORC (Weiner, 2020). Each group of factors was then labeled to represent their key commonality. These are the *synthesized factors (SFs)*.
2. Allocation of SFs to framework constructs: SFs were allocated to TORC-CFIR constructs as described in Table 5. Each SF was assigned to one framework construct only.

**Table 5:** Allocation of SFs framework constructs (synthesis step 2)

Framework construct	Description	TORC or CFIR
*Possible contextual factors*		
Communications	SFs describing information sharing processes across an organization.	CFIR inner setting
Incentive systems	SFs describing incentives and rewards, as well as disincentives or sanctions (e.g., monetary (dis-)incentives, training and career opportunities, recognition within the organization, etc.)	CFIR inner setting
Individuals	SFs describing characteristics of the individuals (e.g., change deliverers, change recipients or other stakeholders) involved in the change implementation process. These may be demographics related to the individual (e.g., age, gender) or to the individual's job (e.g., seniority, specialty, professional role). Further, SFs describing individual perceptions and attitudes, such as self-efficacy, flexibility, and job satisfaction are summarized under this construct.	CFIR individuals
Innovation	SFs describing characteristics of the change to be implemented (e.g., perceived change impact or effectiveness, source of the change, perceived complexity, cost, change adaptability, and topics specific to the change itself, etc.).	CFIR innovation
Organizational culture	SFs describing organizational values, norms and beliefs and their alignment with the change (e.g., patient centeredness, equity, climate, etc.).	TORC
Outer setting	SFs existing outside the organization implementing the change (e.g., critical events, external pressure, local conditions).	CFIR outer setting
Past experience	SFs describing experience with change implementation (e.g., challenges encountered).	TORC
Perceived support	SFs capturing support perceived by organizational members (e.g., financial, emotional, behavioral, or structural support).	TORC
Policies and procedures	SFs describing existing policies and procedures influencing change implementation (e.g., codes of conduct, recruitment policies, regional or national laws).	TORC
Process	SFs describing the process of change implementation (e.g., being in a certain implementation phase, identification of implementation barriers, use of implementation strategies, adaptations to the implementation).	CFIR implementation process
Relational connections	SFs about linking structures between individuals, teams, or organizations (e.g., internal networks).	CFIR inner setting
Structural assets/deficits	SFs describing structural characteristics related to the organization (e.g., size, ownership, population served, number of employees, etc.).	TORC/CFIR inner setting
Trusting relationships	SFs describing the nature of professional relationships within an organization, unit, or team.	TORC
*Change valence*		
Change valence	SFs describing the perceived value of a planned change among organizational members (e.g., due to perceived urgency, anticipated benefit, or perceived importance).	TORC
Mission alignment	SFs characterizing the alignment of the planned change with an organization's overarching goals, plans and commitments.	CFIR inner setting
Tension for change	SFs describing a perceived need for change among organizational members (e.g., because the situation is inacceptable).	CFIR inner setting
*Informational assessment*		
Informational assessment	SFs describing organizational members’ use of information to develop change efficacy judgements. Such information, for example, may include the implementation team's confidence of their ability to implement the change.	TORC
Compatibility	SFs describing the fit between a change and an organization's existing workflows, systems, and processes.	CFIR inner setting
Resource availability	SFs describing the availability of resources—in a broader sense—for implementing a change (e.g., funding, material and equipment, knowledge, space, workforce and their working conditions).	TORC/CFIR inner setting
Situational factors	SFs describing situational factors affecting the change implementation (e.g., situational fluctuation of time available to dedicate to the change, internal climate of support, local conditions or changes affecting the implementation, etc.).	TORC
Task demands	SFs referring to the courses of action required by the change implemented (e.g., task difficulty, task divisibility, task sequence etc.).	TORC

*Note.* TORC = Theory of Organizational Readiness for Change; CFIR = Consolidated Framework for Implementation Research; SFs = synthesized factors.

## Results

### Identified studies

After de-duplication and abstract screening, we assessed 346 full-texts for eligibility. We identified 47 studies for inclusion through database searches (*n* = 45) and bibliography screening (*n* = 2). The PRISMA flowchart (Figure 2) displays the screening process, including reasons for full-text exclusion.

### Study quality

Supplemental Material C presents quality ratings of quantitative studies, divided into quantitative descriptive studies (*n* = 27), nonrandomized studies (*n* = 1), and randomized controlled studies (*n* = 2), as well as quality ratings of mixed-methods studies (*n* = 17), all of which used quantitative descriptive designs.

Among the 30 quantitative studies, only five received “Yes” ratings across MMAT criteria. For most studies (*n* = 18), one or two MMAT criteria were assessed negatively, or could not be assessed at all. This particularly applied to the criteria about nonresponse bias (*n* = 19) and sample representativeness (*n* = 14).

Across the 17 mixed-methods studies, the qualitative elements were generally rated as satisfactory, with 12 of these being positively assessed across all MMAT criteria. One study (Chang et al., 2013) did not receive any positive rating for its qualitative, but solely positive ratings for its quantitative part. The quality of the quantitative sections of these mixed-methods studies was generally assessed as moderate, with only five studies being rated as solely positive across quantitative MMAT criteria. The remaining studies had either three to four (*n* = 9) or one to two (*n* = 3) positive ratings, with information lacking for the same criteria that dominated the quantitative studies: nonresponse bias and sample representativeness. The mixed-methods section was generally rated more critically, with only two studies (Elango et al., 2018; Hearld et al., 2022) achieving a purely positive assessment across all mixed-methods criteria. Further seven studies received three to four positive ratings, and the remaining eight studies had one or two positive ratings. Information was lacking for especially two MMAT criteria: addressing divergencies between qualitative and quantitative results (*n* = 13) and adherence to quality criteria established for the two methodological traditions (*n* = 15).

### Study characteristics

Studies reported on the implementation of various changes across mental, behavioral, physical, and preventive health settings. Table 6 displays the characteristics of included studies, including study country and setting, change implemented, healthcare sector, ORC measures used, and reported ORC results.

**Table 6:** Main study characteristics

First author, publication year	Country	Aim	Implemented change	Healthcare setting	Healthcare field	Individual participants (*n* or n_1_/n_2_)^†^, sites (*N*)	ORC measure (subscales)	Reported factors (RFs) with ORC result	Synthesized factors (SFs)
Abrahamsen et al., 2017	Denmark	To assess readiness for change among staff prior to launching an orthogeriatric unit	A new orthogeriatric unit	Hospital	Orthogeriatric care	*n* = 113 *N* = 1	Adaptation from Kristensen and Nøhr (2000) (knowledge and understanding, need for change, readiness for change, planning for change)	Age: no correlation with ORC Department: no difference in ORC between departments Duration of employment: no correlation with ORC Professional group: ORC was higher in physicians and staff with no patient contact than in nursing staff and therapists Seniority: no correlation with ORC Specialty: ORC was higher in surgeons than in medical practitioners	Individual demographics, job-related demographics, medical specialty, professional role
Adelson et al., 2021	Australia	To report on readiness for change among the midwives, nurses and doctors transitioning to a new model of care (MoC).	Transitioning to a new model of care for pregnant women	Hospital with outpatient clinic	Midwifery	*n* = 56 *N* = 5	ORIC^1^ (change commitment, change efficacy)	Professional group: no differences in ORC between MoC midwives, physicians, nurses, and hospital midwives	Professional role
Akande et al., 2019	Canada	To examine the commitment of the Nunavut Health Department to implementing obesity prevention policies and programs in the Canadian Arctic.	Obesity prevention policies or programs	Public health institution	Obesity prevention	*n* = 67 *N* = 1	Adapted ORIC^1^ (subscales not reported)	Knowledge: positive correlation of some items with ORC Perceived value: strong positive correlation with ORC Resource availability: strong positive correlation with ORC in part of the analyses	Change valence, knowledge, resource availability
Alameddine et al., 2015	Lebanon	To assess readiness of clinical providers to adopt quality indicators.	Primary healthcare quality and safety indicators	Primary healthcare centers	Quality and safety	*n* = 943 *N* = 92	Readiness for Organization Change (ROC) scale^2^ (appropriateness of performance reporting, management support, efficacy, personal valence)	Age: no association with ORC Center ownership: higher ORC in religious centers than in public or NGO centers. Employment status: higher ORC in full-time workers than casual and part-time workers Gender: no association with ORC, except for appropriateness subscale Marital status: no association with ORC Safety attitude: positive association with ORC Type of provider: ORC differed between nurses, specialists, allied health professionals; and family physicians, specialists, dentists and allied health professionals had higher odds for higher efficacy subscale values Years of experience: higher ORC in providers with >15 years of experience than in those with <15 years	Individual attitudes, individual demographics, job-related demographics, organizational demographics, professional role
Becker et al., 2016	USA	To evaluate the implementation effectiveness of a training model among opioid addiction treatment providers.	Contingency management adoption	Satellite clinics	Opioid addiction treatment	*n* = 60 *N* = 15	ORC-S^3^ (motivation for change, adequacy of resources, staff attributes, organizational climate)	The use of a theory-driven implementation strategy: higher ORC when theory-driven implementation strategy was used, but only for adequacy of resources subscale, not for the remaining subscales.	Use of certain implementation strategies
Birnie et al., 2022	Canada	To identify current practices and alignment with pediatric surgical pain care recommendations, as well as health system readiness for change.	Translational pain services	Tertiary/quaternary children's hospitals, rehabilitation hospitals, community/regional hospitals, children's community treatment centers	Pediatric surgical pain services	*n* = 85 *N* = 20	ORIC^1^ (change commitment, change efficacy)	Type of institution: no differences in ORC between tertiary/quaternary hospitals and other institutions Type of population served in the institution: no differences in ORC between institutions that serve pediatric populations only versus institutions that also serve adults	Organizational demographics
Bohman et al., 2008	USA	To determine whether the Medical Organizational Readiness for Change (MORC) was able to identify differences among sites and between staff roles in readiness-to-change dimensions.	Screen, Brief Intervention, Referral to Treatment (SBIRT) program	Community health program clinics, emergency center	Substance abuse	*n* = 184 *N* = 4	MORC^4^, adapted from ORC-S^3^ (need for external guidance, pressure to change, organizational readiness to change, individual readiness to change, workgroup functioning, work environment, autonomy support, and alcohol and drug focus)	Professional role: higher ORC in administrative staff than in clinical staff Type of institution: higher ORC in community health program clinics than in emergency center	Organizational demographics, professional role
Briggs et al., 2022	USA	To understand the acceptability and feasibility of the implementation of tele-dermatology during the COVID-19 pandemic, as well as organizational readiness for the implementation of tele-dermatology.	Implementation of tele-dermatology during the COVID-19 pandemic	Association of professors of Dermatology members	Tele-dermatology	*n* = 35 *N* = unclear	Adapted ORCA^5^ (culture and resources)	Acceptability: lower ORC associated with lower acceptability Plan to use telemetry postpandemic: those with high ORC plan to use telemetry postpandemic Practice type: no association with ORC Race: no association with ORC Sex: no association with ORC Years in practice: no association with ORC	Acceptability, change or intervention-specific topics, individual demographics, job-related demographics, organizational demographics
Burnett et al., 2010*	UK	To investigate the extent to which readiness themes in the literature could be applied.	Implementation of a safer patient initiative (SPI) program	Health service trusts	Patient safety	*n*_1 _= 41 *n*_2 _= 34 *N* = 4	Respondents were asked “How ready was your organization at the start of SPI for successful implementation of the program?” (culture and attitudes towards quality and safety, systems and infrastructure, availability of resources)	Degree to which SPI was driven by top-down or bottom-up approach: negative correlation with ORC Effect of SPI on quality and safety performance in the trust: positive correlation with ORC Success in spreading throughout the organization: positive correlation with ORC Sustainability of SPI benefits: positive correlation with ORC	Effectiveness/impact, sustainability, top-down versus bottom-up approach to implementation
Chang et al., 2013*^,‡^	USA	To improve understanding of the uptake of depression care improvement models by investigating the determinants of adoption of three alternatives, Veterans Affairs system-endorsed approaches to improving routine depression care.	Adoption of a depression care improvement model	Veterans Affairs primary care practices	Depression care	*n*_1_ = unclear *n*_2 _= unclear *N* = 225	ORC^3^ (adequacy of resources, motivation for change, staff attributes, organizational climate)	Implementation of different models with varying complexity: some ORC subscales were associated with the adoption of the model, for example, having psychologist on primary care staff was associated with adoption of Behavioral Health Laboratory model (a more complex model)	Complexity of the change
Chang et al., 2023*	USA	To understand how structured transition processes were operationalized within pediatric rheumatology practices and what factors were perceived to enable adaptations during a global pandemic.	Implementing structured transition policy interventions	Outpatient pediatric rheumatology clinics	Pediatric rheumatology	*n*_1 _= 11 *n*_2 _= 8 *N* = 9	ORIC^1^ (change commitment, change efficacy)	Implementation period: ORC was highest in sites that remained in the preparation phase, and sites that started implementing or withdrew from implementation efforts had low ORC	Implementation phase
Cunha-Cruz et al., 2017	USA	To assess organizational readiness for change before the implementation of system changes within the organization.	Population-centered Risk and Evidence-based Dental Interprofessional Care Team (PREDICT)	Dental care practices	Dental care	*n* = 181 *N* = unclear	ORIC^1^ (change commitment, change efficacy)	Burnout: negative association with ORC (in unadjusted and partially also in adjusted analyses) Job-related stress: partial negative association with ORC in unadjusted analyses, but not when adjusting for demographics/workforce characteristics Job satisfaction: positive association with ORC in unadjusted analyses, but not when adjusting for demographics/workforce Likelihood to leave practice: negative association with ORC in unadjusted analyses, but not when adjusting for demographics/workforce characteristics Office/practice chaos: negative association with ORC (in unadjusted and partially also in adjusted analyses) Organizational climate: positive association with ORC Support for caries arrest treatments: positive association with ORC (in unadjusted and partially also in adjusted analyses) Support for PREDICT: positive association with ORC in unadjusted analyses, but not when adjusting for demographics/workforce characteristics Support for the company's mission: positive association with ORC (in unadjusted and partially also in adjusted analyses) Type of staff member: being a central staff member as opposed to being a dentist or clinic staff was positively associated with ORC in unadjusted analyses, but not when adjusting for demographics/workforce characteristics	Change or intervention-specific topics, job-related stress, job satisfaction, likelihood to leave workplace, organizational climate, professional role, support for the company's mission, support for the change
Dönmez et al., 2020	Turkey	To examine the readiness for health information technologies among medical and administrative staff as well as to evaluate the effects of information security status on readiness.	Use of Health Information Technologies	Training/research/public hospitals, oral and dental health centers	Public health	*n* = 375 *N* = 15	OITIRS^6^ (organizational readiness, technological readiness)	Age: higher ORC in medical staff aged > 45 than in ages 20–24, no association in administrative staff Educational level: higher ORC in medical staff graduated from high school than in those with a bachelor's degree, no association in administrative staff Professional role: no differences in ORC between medical and administrative staff Technology-related items/awareness of importance of information security: positive correlation with ORC for both medical and administrative staff Type of institution: higher ORC in medical staff in oral/dental health centers compared with others, no association in administrative staff	Individual demographics, organizational demographics, professional role, technology-related topics
Elango et al., 2018*	USA	To examine the relationship between the readiness to change of each practice and the success of the intervention, as well as any potentially modifiable factors and external supports affecting readiness to change.	Outpatient antimicrobial stewardship program	Hospital-affiliated network of pediatric primary care practices	Pediatric outpatient primary care	*n*_1 _= 136 *n*_2 _= 31 *N* = 26 (12 for qualitative interviews)	ORCA^5^ (evidence, context, facilitation)	Group cohesion: high ORC practices draw on preexisting shared practice behavior, low ORC sites described others’ behaviors as challenging Group communication with regard to feedback: high ORC practices had regularly exchanging clinicians, leading to open feedback, and low ORC practices described limited communication Group process for change (proactive vs. passive effort to improve): high ORC practices rather took a proactive approach to problem-solving, and low ORC practices described a more passive engagement	Communication, proactive versus passive effort to improve, preexisting shared practice behavior
Gallant et al., 2023*^,‡^	Unclear	To use the CFIR and Unified Theory of Acceptance and Use of Technology in the identification of determinants to maximize the likelihood of successful implementation of an automated pain behavior monitoring system in Long-term care settings.	ABPM system implementation	Long-term care facilities	Pain behavior monitoring	*n*_1 _= 157 *n*_2 _= 74 *N* = 2 (for qualitative part, *N* = unclear for quantitative part)	ROC^2^ (appropriateness, personal valence, management support, change efficacy)	Effort expectancy: mediated the relationship between ORC subscales management support/appropriateness and behavioral intentions, but not the remaining subscales Facilitative conditions: did not mediate the relationship between ORC and behavioral intentions Performance expectancy: mediated the relationship between ORC subscales management support/change efficacy and behavioral intentions, but not the remaining subscales Social influence: did not mediate the relationship between ORC and behavioral intentions	Effectiveness/impact, situational factors, social influence, task demands
Garner et al., 2022	USA	To test if Positive Health Check (PHC) supports viral suppression and retains people with HIV in care.	PHC	Primary care practices and ambulatory clinics	HIV prevention	*n*_1 _= 126 *n*_2 _= 126 *N* = 4	ORIC^1^ (change commitment, change efficacy)	Communication: when ORC was low, there was some frustration due to indirect communication Engagement: high ORC in clinics that showed early engagement Organizational implementation challenges: turnover, disintegrated workflows and lack of confidence in navigating challenges were noted along with low ORC	Communication, engagement, organizational implementation challenges
Geerligs et al., 2021*	Australia	To assess organizational readiness for change at commencement of the Australian clinical pathway for the screening, assessment and management of anxiety and depression in adult cancer patients (ADAPT CP) implementation, identify factors associated with differences in levels of organizational readiness across services and identify factors specific to the introduction of a psycho-oncology intervention.	ADAPT CP	Unclear	Psycho-oncology	*n*_1 _= 65 *n*_2 _= 44 *N* = 6	ORIC^1^ (change commitment, change efficacy)	Beliefs regarding the efficacy and sustainability: associated with high ORC sites Engagement with the preimplementation process: high ORC sites developed a sense of ownership preimplementation, medium ORC sites felt imposition Factors specific to psycho-oncology: concerns about mental health literacy and psycho-oncology clinical pathways decreased ORC Flexibility: high ORC associated with flexible behaviors and responsibilities, medium ORC associated with concerns related to extra tasks Implementation preparation: High ORC sites anticipated barriers and were confident in navigating these, medium ORC sites had concerns about resolving potential issues Workplace culture: high ORC in sites that have collaborative, proactive, supportive culture. Medium ORC in sites with a greater sense of fragmentation.	Change or intervention-specific topics, engagement, flexibility to change behaviors, implementation preparation, organizational culture, sustainability
Goebel et al., 2020*	USA	To implement a scalable version of the “Kicking Catheter-Associated Urinary Tract Infection Campaign” across four geographically diverse veterans affairs (VA) facilities, with four control sites.	guideline-concordant management of asymptomatic bacteriuria; improvement of antibiotic prescriptions	Inpatient medicine units and long-term care units within VA medical centers	Antimicrobial stewardship	*n* = 104 *N* = 4	Adapted ORCA^5^ (evidence, context)	Healthcare professional type: no difference in ORC, except for the culture subscale, in which pharmacists had lower scores than providers	Professional role
Guerrero et al., 2020	USA	To explore the relationship between inner setting characteristics of the emergency department (e.g., leadership, readiness for change, organizational climate) and practitioner support for Opioid Use Disorder (OUD) treatment and attitudes towards people with OUD.	Monitoring-assisted treatment for OUD Treatment (MAT)	University hospital	OUD treatment and emergency care	*n* = 241 *N* = 1	Attitude toward change instrument^7^ (subscales not reported)	Optimism to treat people with OUD: no association with ORC Professional role: no differences in ORC between managers, physicians, nurses, or other staff Self-efficacy to treat OUD: no association with ORC Stereotypes of drug users: no association with ORC Support for best practices to treat OUD: positive association with ORC Support for MAT: positive association with ORC	Change or intervention-specific topics, individual attitudes, professional role, self-efficacy, support for the change
Harrison et al., 2022	Australia	To establish the level of individual and collective change readiness among healthcare staff, and to establish whether there is an association between affective commitment to change and change self-efficacy and change readiness.	Virtual pharmacy services, in-home monitoring services, emergency department expansion, and an outpatient administration redesign.	Local health districts, one servicing a metropolitan region and one servicing a rural/remote region	Virtual pharmacy services, in-home monitoring services, emergency department expansion, and an outpatient administration redesign	*n* = 30 *N* = unclear	Self-developed, validated elements of a survey (subscales not reported)	Affective commitment: positive association with ORC, also when controlling for change self-efficacy Change self-efficacy: positive association with ORC, became negligible after controlling for affective commitment Individual change readiness: positive association with ORC	Commitment, individual readiness for change, self-efficacy
Hearld et al., 2022*	USA	To identify and tailor preferred implementation strategies (e.g., education, feedback) to each clinic in support of their efforts to “hardwire” the decision-aid into routine care processes.	An electronic decision-aid designed to educate lupus patients	Rheumatology clinics	Rheumatology	*n*_1 _= 135 *n*_2 _= 88 *N* = 15	ORIC^1^ (change commitment, change efficacy)	Average years in clinic: more years of experience correlated with lower ORC Clinic size: no association with ORC Culture: association between rational culture and lower ORC Medical specialty: no difference in ORC between clinics with rheumatology specialty only and clinics with multiple specialties Number of locations: no association with ORC Ownership: higher ORC in nonuniversity owned clinics than university-owned clinics	Job-related demographics, medical specialty, organizational culture, organizational demographics
Hoffmann et al., 2022	Germany	To assess the association of personal (technology acceptance) and organizational (innovation climate) factors on the readiness for the implementation of a webcam system in neonatal intensive care units (NICUs) from the perspective of lead nurses and physicians.	NeoCamCare, a publicly funded project that evaluates webcam use in NICUs	NICUs	Neonatal intensive care	*n* = 234 *N* = unclear	Organizational Change Questionnaire^8^ (Readiness for Change—emotional and intentional readiness)	Age: no association with ORC Gender: no association with ORC Innovation climate: no association with ORC Professional role: no difference in ORC between nurses and physicians Technology acceptance: associated with higher ORC in physicians, no association with ORC in nurses	Implementation climate, individual demographics, professional role, technology-related topics
Jakobsen et al., 2020	Denmark	To investigate the effect of a participatory organizational intervention on social capital and organizational readiness for change.	Improving the use of assistive devices	Hospitals	Patient handling	*n* = 625 *N* = 5	ORIC^1^ (change commitment, change efficacy)	Use of a participatory intervention: there was no difference in ORC between groups over time, but the group randomized to the participatory intervention showed higher ORC improvement from baseline to follow-up than the control group.	Use of certain implementation strategies
Joudrey et al., 2020*	USA	To develop a comprehensive understanding of barriers and facilitators of Medication for Alcohol Use Disorder (MAUD) adoption among hospital-based health professionals who may routinely participate in care for patients with alcohol use disorder (AUD).	adoption of MAUD among hospital-based health professionals	Academic medical center	AUD care	*n* = 57 *N* = 1	Adapted ORCA^5^ (evidence, context)	Prescribing status: no difference in ORC between prescribers and non-prescribing professions, except for the availability of resources subscale, where prescribers had lower ORC levels than non-prescribing professions.	Professional role
Kujala et al., 2019	Finland	To examine whether the health care leaders’ positive expectations of a patient portal and perceptions of its implementation are associated with their own and the subordinate professional support for the portal in the preimplementation phase.	Providing a national patient portal for self-management and self-service.	Primary care health center, hospital, psychiatric outpatient clinic, elementary school health care, emergency care, dental care, and other	Patient self-assessment and symptom management across different care specialties	*n* = 2468 *N* = 44	Organizational readiness scale^9^ (subscales not reported)	Leadership support for the patient portal: positive association with ORC	Leadership support
Le et al., 2021	USA	To evaluate knowledge change after provision of tobacco education training to employees at substance use treatment centers (SUTCs) and the organizational-level factors impacting knowledge change.	Taking Texas Tobacco Free	SUTCs	Tobacco use	*n* = 580 (pre)/525 (post) *N* = 19	Adapted ORIC^1^ (resource availability, change efficacy, change valence, change commitment, task knowledge)	Knowledge gain from pre- to posttraining: ORC moderated training effectiveness, such that sites with higher ORC demonstrated higher knowledge gain from pre- to posttraining	Effectiveness/impact
Lundgren et al., 2012*	USA	To examine the association between clinical staff and program director ratings of the organizational capacity of their treatment unit and the level of barriers experienced when implementing a new evidence-based practice (EBP).	Implementing one EBP, of which the most common ones were motivational interviewing, adolescent community reinforcement approach, assertive community treatment, and cognitive behavioral therapy.	SUTCs	Addiction treatment	*n*_1 _= 806 *n*_2 _= 806 *N* = 330	TCU ORC^10^—staff and director versions (motivation for change, adequacy of resources, staff attributes, and organizational climate)	Level of barriers for implementing an EBP: among staff and program directors, high ORC was partially (in some subscales) associated with higher levels of reported barriers	Level of barriers
Lundgren et al., 2013*	Unclear	To examine whether staff perceptions of both their unit's organizational readiness for change and level of barriers experienced when implementing a newly funded EBP are associated with the level of modifications made to the EBP in the implementation process.	Implementing one EBP, of which the most common ones were motivational interviewing, adolescent community reinforcement approach, assertive community treatment, and cognitive behavioral therapy.	SUTCs	Addiction treatment	*n*_1 _= 510 *n*_2 _= 510 *N* = 330	TCU ORC^10^-staff (motivation for change, adequacy of resources, staff attributes, and organizational climate)	Level of modifications made to EBP: association with some ORC subscales	Level of modifications made to the change
Messer et al., 2012*	USA	To assess and enhance organizational readiness to adopt information technology, develop a Regional Health Information Organization (RHIO) to share electronic data between medical and ancillary care providers, implement the RHIO and begin active information exchange and evaluate the effect of the intervention on provider-related attitudes and satisfaction with information exchange.	Implementing an integrated IT network within a preexisting community of human immunodeficiency virus (HIV) medical and ancillary care providers	Academic medical center and acquired immunodeficiency syndrome service organizations	HIV care	*n*_1 _= 39 *n*_2 _= unclear *N* = 5	ORC^3^ (ORC-SA and ORC-S, [motivation for change, adequacy of resources, staff attributes, and organizational climate])	Agency type: consistent ORC across medical care providers and ancillary service providers Respondent type: consistent ORC across staff and directors	Medical specialty, professional role
Myers et al., 2017	South Africa	To explore factors associated with readiness to adopt a performance measurement system among substance abuse treatment providers.	Service Quality Measures (SQM) initiative	Residential and outpatient substance abuse disorder treatment facilities	Substance abuse disorder treatment	*n* = 81 *N* = 13	Survey about readiness to adopt SQM initiative^11^ (subscales not reported) and TCU ORC^10^ (pressure to change)	Caseload: positive association with ORC Experience in years: no association with ORC Gender: no association with ORC Role: no association with ORC (directors/counsellors/support staff) Time in current job in years: no association with ORC	Individual demographics, job-related demographics, professional role
Peracca et al., 2021*^,‡^	USA	To understand the impact of mobile tele-dermatology apps on improving access.	VA Telederm application	VA facilities	Dermatology	*n*_1 _= 8 *n*_2 _= 12 *N* = 3	ORIC^1^ (change commitment, change efficacy)	Change valence: not reported Leadership support: not reported Resource availability: not reported Situational factors: not reported	Change valence, leadership support, resource availability, situational factors
Peracca et al., 2023*	USA	To assess VA's readiness to implement the My VA Images (MVAI) app and factors influencing organizational readiness for this type of direct-to-patient tele-dermatology at three VA facilities where the app was first released.	MVAI app	VA facilities	Dermatology	*n*_1 _= 16 *n*_2 _= 17 *N* = 3	ORIC^1^ (change commitment, change efficacy)	Change valence: partially positive impact on ORC: Clinical champion present: partially positive impact on ORC Concerns about clinicians lacking app knowledge: partially negative impact on ORC Concern about finding eligible veterans: partially negative impact on ORC Concern about patient interest: partially negative impact on ORC Concern about workflow disruption or added time/workload: partially negative impact on ORC Confident that facility can implement app: partially positive impact on ORC Facility leadership goal to expand telehealth: partially positive impact on ORC Fits with facility's approach to patient care: partially positive impact on ORC Lack of technical literacy: partially negative impact on ORC Leadership support: partially positive impact on ORC Need more provider buy-in: partially negative impact on ORC Resource availability: not reported Situational factors: not reported Staff commitment: partially positive impact on ORC Strong tele-dermatology program: partially positive impact on ORC Task demands: not report Veteran supportive and willing to spend time to use apps: partially positive impact on ORC Wait and see attitude: partially negative impact on ORC	Change aligning with leadership goals, change valence, commitment, confidence that the facility can implement the change, job-related stress, individual attitudes, leadership support, need of more provider buy-in, patient-related topics, presence of a clinical champion, resource availability, situational factors, strong program already existing, support for the change, task demands, technology-related topics, change fits with the facility's approach to patient care
Pinto et al., 2011	UK	To evaluate the influence of broad situational factors on the perceived success of the SPI program using exploratory regression analysis.	SPI	National Health Service organizations	Patient safety	*n* = 635 *N* = 20	Self-developed (“How ready do you think your organization was for the SPI before it started?,” followed by the presentation of eight items^12^)	Perception of SPI impact: positive association with ORC	Effectiveness/impact
Randall et al., 2020	USA	To assess organizational readiness to implement management/procedural changes related to delivery of evidence-based dental care, and to determine personal, workforce-related, and perceived work environment-related factors associated with readiness.	Oral Health Equity for Alaska	Dental program at a large health care organization	Dental care	*n* = 78 *N* = 3	ORIC^1^ (change commitment, change efficacy)	Job role: partially positive association with ORC Job satisfaction: positive association with ORC Organizational context and resources: positive association with ORC Organizational functioning: positive association with ORC	Job satisfaction, organizational context and resources, organizational functioning, professional role
Rodriguez et al., 2016	USA	To examine the extent to which better teamwork mediated relationships between team member availability and readiness for change	Including interdisciplinary teams for diabetes care	Community health center	Diabetes care	*n* = 619 *N* = 34	Organizational Readiness for Change Assessment instrument^13^ (change culture)	Access to interdisciplinary expertise: positive association with ORC among primary care physicians (PCP), but not in staff members, neither across groups Gender: no association with ORC Race: In the lowest ORC quartile, there were more Latino and non-Latino White PCP compared to Asian and other respondents Team member availability: no association with ORC for PCP, positive association with ORC for staff members and across groups Team size: for staff members, second lowest quartile of team size had lower ORC, but no association with ORC in PCP, neither across groups Teamwork: positive association with ORC for both PCP and staff members, and across groups	Access to interdisciplinary expertise, individual demographics, team member availability, team size, teamwork
Saleh et al., 2016	Lebanon	To assess the readiness of healthcare providers working in primary healthcare centers across Lebanon to use eHealth tools and applications.	eHealth tools	Primary healthcare centers	Primary care	*n* = 213 *N* = 22	ROC^2^ (appropriateness, management support, change efficacy, personally beneficial)	Age: no difference in ORC between age groups Comfort using computers: Gender: no difference in ORC between genders Employment status: Level of education: no difference in ORC between levels of education Occupation: partially positive association with ORC between certain occupational groups Total years of practice: no difference in ORC Total years of practice at respective primary healthcare center: no difference in ORC Sharing computers with colleagues at the respective primary healthcare centers: no difference in ORC	Individual demographics, job-related demographics, professional role, resource availability, technology-related topics
Scales et al., 2017	USA	To examine nurse and medical provider attitudes toward and perspectives on antibiotic stewardship, and how they relate to individual and organizational factors.	Antibiotic stewardship intervention	Nursing homes	Antibiotic stewardship in nursing homes	*n* = 232 *N* = 31	Adapted ORIC^1^ (change commitment, change efficacy)	Having a subspecialty or not: those with subspecialty had higher ORC Primary position of the nursing respondent: partial positive association with ORC, with directors of nursing and other leadership roles having higher ORC than floor nurses Professional group: different ORC between nurses and medical providers	Professional role
Shrubsole et al., 2022*	Australia	To investigate whether improvements in acute speech pathologists’ aphasia management practices were sustained following implementation, whether postimplementation improvements in the rated behavior change domains (barriers) targeted by the implementation intervention were sustained, to determine what factors may have influenced sustainability, including organizational and clinician-level factors and to explore potential explanatory connections between the sustainment outcomes and sustainability factors.	implementing either written information provision or collaborative goal setting	Speech language therapist departments in hospitals	Speech language therapy	*n* = 35 *N* = 4	ORIC^1^ (change commitment, change efficacy)	Implementation intervention that aimed to improve their practice in either written information provision (Intervention A) or collaborative goal setting (Intervention B): no difference in ORC between the intervention clusters	Use of certain implementation strategies
Smelson et al., 2022	USA	To study the impact of the implementation facilitation strategy to support Maintaining Independence and Sobriety through Systems Integration, Outreach and Networking-Veterans Edition (MISSIONVet) implementation and fidelity.	MISSIONVet	VA medical centers	care of homeless veterans with co-occurring mental health and substance abuse disorders	*n* = 77 *N* = 2	Adapted ORCA^5^ (context)	Age: no association with ORC Duration of employment with the VA: partially positive association with ORC Sex: no association with ORC Staff type: no differences in ORC between case managers and peer specialists	Individual demographics, job-related demographics, professional role
Spalluto et al., 2021	USA	To evaluate organizational readiness for change and change valence among clinical providers, staff, and administrators affiliated with radiology and primary care at a single VA medical center.	Lung-cancer screening of high-risk individuals	VA medical center	Lung cancer screening	*n* = 269 *N* = 1	ORIC^1^ (change commitment, change efficacy)	Profession: no differences in ORC between staff, providers, and administrators Self-reported leaders versus nonleaders: higher overall ORC in leaders than in nonleaders Service line: differences in overall ORC between radiology service and primary care	Medical specialty, professional role, leaders versus nonleaders
Stadnick et al., 2022	USA	To examine the preimplementation organizational context including healthcare organizational type, implementation climate, readiness for implementing change of a pediatric integrated care model in pediatric primary care settings.	Access to Tailored Autism Integrated Care (ATTAIN)	Primary care organizations	Pediatric integrated care (autism spectrum disorders)	*n* = 36 *N* = 3	Adapted ORIC^1^ (change commitment, change efficacy)	Implementation climate: Positive association with ORC	Implementation climate
Stanhope et al., 2019*	Unclear	To utilize quantitative data to examine the trajectory of organizational readiness to change and leadership behaviors over a 12-month training initiative and to utilize qualitative data to provide insight into the trajectory of organizational readiness to change and leadership behaviors from the provider perspective.	Person-centered care planning (PCCP)	Community mental health clinics	Mental health	*n*_1 _= unclear *n*_2 _= 104 *N* = 7	Self-developed (single-item 10-point Likert scale [1 = *low*; 10 = *high*] in which consultants made a global assessment of a clinic's readiness to implement the PCCP intervention	Implementation leadership: positive association with ORC Leadership attendance in technical assistance calls: positive association with ORC	Implementation leadership, leadership attendance
Von Treuer et al., 2022	Australia	To investigate whether training in change processes (focusing on transformational leadership and work environment) can increase levels of readiness for change in residential aged care, and whether the increased levels of change readiness can be sustained up to 6 and 12 months after training.	A novel program designed to prepare staff for organization changes in policies and practices to better support aged care residents	Residential aged care facilities	Residential aged care	*n* = 129 *N* = 16	ROC^2^ (appropriateness of performance reporting, management support, efficacy, personal valence)	Being randomized to training versus waitlist-control: At some timepoints, training condition partially showed higher ORC than waitlist control group	Use of certain implementation strategies
Washington et al., 2018	USA	To examine barriers and facilitators to Chronic Disease Self-Management Program (CDSMP) implementation by dialysis facilities, as well as readiness for change factors among dialysis facility staff.	CDSMP	Outpatient dialysis facilities	Outpatient dialysis care	*n* = 63 *N* = 3	ORIC^1^ (change commitment, change efficacy) + one global rating question (“How ready is your facility to implement this program?”)	Age: no association with global ORC, but positive association with ORC measure Average hours worked per week: no association with global ORC, nor with ORC measure Length of time worked at any facility: no association with global ORC, nor with ORC measure Length of time worked at current facility: no association with global ORC, nor with ORC measure Level of education: no association with global ORC, but those with technical, trade school, and college or associate degrees scored higher in parts of the ORC measure than those with a high school, bachelor's or graduate's degree Profession: no association with global ORC, but patient care technicians and nurses scored higher in parts of the ORC measure than social workers, dieticians and other professionals Race: no association with global ORC, nor with ORC measure	Individual demographics, job-related demographics, professional role
Williams et al., 2014	USA	To describe the characteristics of community behavioral health organizations and community health centers and decisionmakers within these organizations prior to deciding about the adoption of Motivational Interviewing (MI). This study also seeks to identify differences between the characteristics of community behavioral health organizations and community health centers, and differences between directors and practitioners within these organizations.	MI	Community health centers, community behavioral health organizations	Mental and behavioral healthcare	*n* = 311 *N* = 92	TCU ORC—staff and director versions^10^ (motivation for change, program resources, staff attributes, organizational climate) and adapted Organizational Readiness and Capacity assessment^14^ (clients, leadership/clinicians/staff, supervision, internal and external stakeholders, program/culture/services, finance and administration, education, and technology)	Role: practitioners reported lower ORC than directors Type of institution: overall, no differences in ORC between community health centers and community behavioral health organizations	Organizational demographics, professional role
Zapka et al., 2013*	USA	To profile the contextual factors associated with readiness to participate in an innovative telemedicine project designed to improve care for patients presenting at rural hospitals with high acuity conditions—sepsis and trauma.	Critical Care Excellence in Sepsis and Trauma program (CREST)	Emergency departments (EDs) within rural hospitals	Telemedicine, sepsis, and trauma	*n*_1 _= 86 *n*_2 _= 23 *N* = 4	Self-developed (“ED staff is receptive to CREST”)	ED culture: contributed little to explaining ORC variation Hospital QI culture: no association with ORC Need for CREST: positive association with ORC Resource perceptions: positive association with ORC Task demands: positive association with ORC	Organizational culture, resource availability, task demands
Zullig et al., 2013	Tanzania	To assess shared beliefs among stakeholders in the organizational ability to initiate and maintain cancer registration activities at a tertiary referral medical center in Moshi, Tanzania.	Implementation of a cancer registry	Tertiary medical center	Cancer registration	*n* = 52 *N* = 1	Unclear, but subscales mentioned are change commitment, change efficacy, determinants of change efficacy	Professional role: no differences in ORC between physicians and administrators Medical specialty: partially different ORC between surgery and internal medicine	Professional role, medical specialty

*Note.* ^+ ^= in mixed methods studies, *n*_1 _= quantitative sample size, *n*_2 _= qualitative sample size; * = mixed methods study; ^‡^ = study investigating RQ2, that is, factors investigated as possible mediators or moderators of ORC and implementation.

^1^ ORIC = Organizational Readiness for Implementing Change Scale (Shea et al., 2014).

^2^ ROC = Readiness for Organization Change (Holt et al., 2007a).

^3^ ORC = Organizational Readiness for Change Scale, the ORC-SA was designed for social service agencies and the ORC-S was designed for substance abuse treatment agencies (Lehman et al., 2002).

^4^ MORC = The Medical Organizational Readiness for Change (Bohman et al., 2008).

^5^ ORCA = Organizational readiness to change assessment instrument (Helfrich et al., 2009).

^6^ OITIRS = Organizational Information Technology Innovation Readiness Scale (Snyder-Halpern, 2002), of which only organizational readiness scale is considered for this SLR.

^7^ Dunham-Presenter et al. (1989).

^8^ Bouckenooghe et al. (2009).

^9^ Paré et al. (2011).

^10^ TCU ORC = Texas Christian University Organizational Readiness for Change scales (Lehman et al., 2002).

^11^ Items not reported, Myers et al. (2017).

^12^ Scale items of the self-developed measure included “recognition of existing safety problems,” “knowledge of how to tackle safety problems,” “systems and infrastructure to support safety improvement” (Pinto et al., 2011).

^13^ Source of instrument not reported.

^14^ Allred et al. (2005). Lundgren et al. (2013) builds on the previously conducted study by Lundgren et al. (2012). Peracca et al. (2021) and Peracca et al. (2023) are both studies as part of a larger trial (Done et al., 2018).

### Research questions

Out of 47 included studies, 44 reported on factors falling under RQ1, that is, factors investigated in relation to ORC (e.g., Adelson et al., 2021; Saleh et al., 2016). Two studies investigated factors related to RQ2, that is, factors investigated as possible mediators or moderators of ORC and implementation (i.e., Chang et al., 2013; Gallant et al., 2023). For example, Chang et al. (2013) investigated the relation between ORC and the implementation of depression care models and found that some ORC subscales were related to the adoption of these models. Gallant et al. (2023) explored mediators between ORC and behavioral intentions to adopt an automated pain management monitoring system and found a partially mediating effect of effort and performance expectancy between ORC and the intention to adopt this system. One study RFs matching both RQs (Peracca et al., 2021). The RFs and SFs investigated in relation to RQs are listed in Table 6. A detailed account of SFs is provided in Supplemental Material D.

### Study design and methodology

Forty-one studies used a nonexperimental or observational design, four used an experimental design, and two a hybrid design. Thirty studies used quantitative methods, whereas 17 studies employed mixed methods.

#### Study settings

Studies were conducted in a broad range of in- and out-patient settings. Health care organizations included acute care hospitals (regional, tertiary, pediatric, and university hospitals) or specific units within hospitals, such as neonatal intensive care units or emergency departments. Furthermore, outpatient studies were conducted in specialized clinics, primary care practices and centers, community health centers, substance use treatment centers, long-term care facilities, nursing homes, and dental care practices, among other settings. Different types of Veterans Affairs facilities were used in studies conducted in the United States.

#### ORC measures used

Various quantitative ORC measures (or adaptations thereof) were administered in included studies. Used most frequently was the ORIC (18 studies) developed based on the TORC by Shea et al. (2014). Five studies used the ORCA (Helfrich et al., 2009), and four each used the Readiness for Organization Change (ROC; Holt, Armenakis, Feild, & Harris, 2007), ORC (Lehman et al., 2002), and Texas Christian University ORC (TCU ORC; Lehman et al., 2002) scales. One study measured ORC with the Organizational Information Technology Innovation Readiness Scale (Snyder-Halpern, 2002). The remaining 11 studies used self-developed items, adaptations from existing surveys, or did not clearly report their ORC measures. Table 6 displays ORC measures used for each study.

#### Timing, frequency and level of ORC measurements

The timing and frequency with which ORC was measured are visualized in Table 7. Nineteen studies measured ORC before implementation only, eight studies reported having measured ORC during implementation only, and four studies measured ORC retrospectively, after implementation only. Three studies measured ORC before and during implementation, and three measured ORC before, during, and after implementation. The timing of ORC measurement was unclear in ten studies. The number of ORC measurements within a study ranged from one to 12 across 46 studies, with 40 reporting single ORC measurements. The frequency with which ORC was measured was unclear in one study.

**Table 7:** Timing and frequency of ORC measurement per study.

Study	Before implementation	During implementation	After implementation	Number of ORC^1^ measurements
Abrahamsen et al. (2017)				1
Adelson et al. (2021)				1
Akande et al. (2019)	*Unclear*			1
Alameddine et al. (2015)	*Unclear*			1
Becker et al. (2016)				3
Birnie et al. (2022)				1
Bohman et al. (2008)				1
Briggs et al. (2022)				1
Burnett et al. (2010)				1
Chang et al. (2013)				1^a^
Chang et al. (2023)				1^b^
Cunha-Cruz et al. (2017)				1
Dönmez et al. (2020)	*Unclear*			1
Elango et al. (2018)				1
Gallant et al. (2023)				1
Garner et al. (2022)				8
Geerligs et al. (2021)				3
Goebel et al. (2020)				1
Guerrero et al. (2020)	*Unclear*			1
Harrison et al. (2022)				1
Hearld et al. (2022)	*Unclear*			1
Hoffmann et al. (2022)	*Unclear*			1
Jakobsen et al. (2020)				3
Joudrey et al. (2020)				1
Kujala et al. (2019)				1
Le et al. (2021)				1
Lundgren et al. (2012)				1
Lundgren et al. (2013)				1
Messer et al. (2012)				1
Myers et al. (2017)				1
Peracca et al. (2021)				*unclear*
Peracca et al. (2023)	*Unclear*			1
Pinto et al. (2011)				1
Randall et al. (2020)	*Unclear*			1
Rodriguez et al. (2016)				1
Saleh et al. (2016)				1
Scales et al. (2017)				1
Shrubsole et al. (2022)				1
Smelson et al. (2022)				1
Spalluto et al. (2021)				1
Stadnick et al. (2022)				1
Stanhope et al. (2019)	*Unclear*			12
Von Treuer et al. (2022)				4
Washington et al. (2018)	*Unclear*			1
Williams et al. (2014)				1
Zapka et al. (2013)				1
Zullig et al. (2013)				1

*Note.* ^1^ORC = Organizational Readiness for Change. Light gray cells indicate that the timing of ORC measurement varied across the study sites.

^a^Some sites were planning to implement, some sites already did implement the change.

^b^Some were preparing for implementation, some had already begun implementing, and some had withdrawn by the time of the readiness assessment.

With ORC representing a collective rather than an individual construct, all studies measured ORC at the organizational level, as specified by the respective ORC measures used. Additionally, five studies further indicated team-level considerations in their ORC measurements. For example, Akande et al. (2019) worded items as referring to a group (e.g., “we know…” instead of “I know…”) to intentionally emphasize a team perspective in their ORC measurement. Further examples are shown in Supplemental Material E.

### Synthesized factors

**Figure 3:**
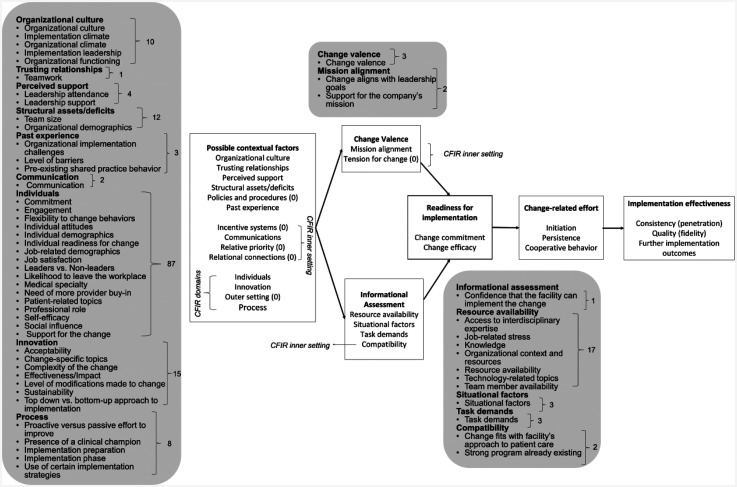
Synthesized factors (SFs) assigned to TORC-CFIR framework

*Note.* White, rectangular boxes represent the TORC constructs with indicated CFIR components and gray boxes represent SFs assigned to the according TORC-CFIR framework constructs. The numbers describe the number of entries within a TORC or CFIR construct (e.g., number of times an SF was represented across included studies). TORC = Theory of Organizational Readiness for Change; CFIR = Consolidated Framework for Implementation Research.

Figure 3 portrays a detailed landscape of SFs investigated in combination with ORC or investigated as potential mediators or moderators of the relationship between ORC and implementation, mapped to the TORC-CFIR framework. Overall, Figure 3 reflects that most SFs identified relate to the *possible contextual factors* component of the TORC-CFIR framework. Less prominently reported were SFs related to *informational assessment*, followed by those falling under *change valence*.

Prominent among *possible contextual factors* are individual traits investigated in relation to ORC. These range from individual- (e.g., age, gender) and job-related demographics (e.g., seniority, years of experience) to psychological constructs (e.g., attitudes, self-efficacy). SFs related to the implemented *innovation* also had a focal role, with innovation acceptability, complexity, sustainability, and perceived effectiveness being central examples. Conversely, there was a notable absence of *possible contextual factors* for the TORC element *policies and procedures* and the CFIR domain *outer setting*, both of which are related. Furthermore, the CFIR *inner setting* constructs *incentive systems*, *relative priority,* and *relational connections* lack representation across included studies.

Within the TORC determinant *informational assessment*, *resource availability* has a striking presence among included studies, with most SFs belonging here, ranging from general availability of resources to more specific resources such as knowledge, team member availability, and access to interdisciplinary expertise.

The TORC *change valence* determinant includes SFs about change valence itself, and SFs about mission alignment. Contrary to *informational assessment*, *change valence* is underrepresented among the SFs investigated in combination with ORC in healthcare studies.

## Discussion

With this SLR, we provide an overview of factors examined in relation to ORC and of potential relevance for explaining implementation results in healthcare studies, be these positive, negative, or null findings. We synthesized the empirical healthcare literature on factors investigated in relation to ORC, including possible mediators or moderators of the relationship between ORC and implementation. Forty-seven studies were included in this review. Factors were mostly investigated *in relation to* ORC rather than as possible *mediators* or *moderators* of the relationship between ORC and implementation. The limited number of studies linking ORC to implementation identified with this SLR confirms the previously critiqued shortage of studies examining ORC prospectively. In the context of the TORC-CFIR framework that guided this SLR (Figure 1), most factors reported were *possible contextual factors*, with the focus being on individual traits and the innovation itself. Less examined was the *informational assessment* determinant, with most factors representing aspects of resource availability. Least represented was the ORC determinant *change valence*.

Our results show that an extensive variety of factors has been researched in relation to ORC, with only few factors investigated across multiple studies, and many factors having been the focus of a single or few studies only. These factors are similar, but due to the use of slightly different concepts, they are assigned to different TORC-CFIR framework elements. The diversity of ORC conceptualizations and theories, in combination with this somewhat diffuse landscape of factors synthesized from the empirical literature, highlights the need to enhance the conceptual clarity surrounding ORC (Holt, Armenakis, Harris, & Feild, 2007; Kelly et al., 2017).

The knowledge base on whether and how *change valence* influences ORC remains unclear. While the implicit assumption that ORC may be stronger if organizational members value an intended change may seem intuitive, ORC studies focusing on this aspect are scarce. One reason may be that some scholars view change valence as an ORC *component* (e.g., Armenakis et al., 1993), whereas change valence is conceptualized as an ORC *determinant* in the TORC guiding this SLR (e.g., Weiner, 2020). Viewing change valence as a determinant or a component of ORC is a substantial conceptual difference—highlighting the importance of transparent theory use and conceptualization when measuring ORC. To set the conceptual boundaries of the theory and measure used, a clear description of the underlying constructs measured as part of an ORC assessment is indispensable.

Simultaneously, these findings illustrate two major obstacles to advancing the field. First, as already highlighted, lacking consensus about core ORC components hinders attempts to clearly define ORC and its conceptual boundaries. This may be due to the common use of “readiness” in everyday language (Weiner, 2020). Hence, scholars may mistakenly assume a preexisting shared understanding of “organizational readiness for change.” Validation studies of ORC measures are one attempt to create definitions of ORC. However, the plethora of available measures mirrors insufficient uniformity in ORC definition and conceptualization.

This lack of conceptual clarity exacerbates the second obstacle to advancing ORC research—the need for more nuanced theorizing about *why* certain implementation results occur, how ORC might contribute to such results, and about causal mechanisms between implementation determinants, strategies, and proximal and distal outcomes (Lewis et al., 2022, 2018). More nuanced, causal theories will help identify what might influence ORC, for example, in the form of national or regional culture, organizational structures, or team-level dynamics, and why these factors have such influences.

Most factors categorized in this SLR as *possible contextual factors* were defined at the individual level. This mirrors a tendency in ORC research to examine individual factors with the aim to understand ORC. This contrasts with the notion of ORC as a collective construct (Scaccia et al., 2015; Weiner et al., 2020) rather than an individual trait. It also warrants caution, as social scientists have long discussed the problem of data aggregation to a higher unit of analysis when researching multidimensional constructs (Chan, 2019), such as ORC. In healthcare settings, change is frequently implemented at a collective level, demanding behavior change from various individuals to achieve anticipated benefits (Michie et al., 2018). Hence, different roles with interdependent responsibilities contribute to the implementation effort, which can be analogized as a “team sport.” This implies that the sum of single individuals changing behaviors will not be sufficient for an organization to fully establish collective change (Weiner, 2020), suggesting that ORC cannot be sufficiently captured by aggregating individual-level measurement.

Furthermore, insights into how individual factors connect to ORC are only meaningful if they are of practical relevance, that is, can be leveraged by an organization when implementing change. Individual factors that are rarely modifiable have limited practical relevance (e.g., personality traits, attitudes, personal values), whereas others (e.g., staff seniority, job position) are within organizational control and may be investigated. Therefore, when implementing collective behavior change interventions, there may be value in examining higher-level dynamics and characteristics, either on the team (e.g., team member composition, teamwork) or organizational level (e.g., formal recognition of workflows, hierarchical structures), as implementation teams or the organization can influence these (Rafferty et al., 2013; Weiner, 2020). Team research offers various conceptual frameworks (i.e., composition models) specifically addressing issues related to measurement across multiple levels of analysis (Chan, 1998, 2019).

The authors of a recent review mapping items of ORC assessments to the CFIR (Miake-Lye et al., 2020) suggested capturing the team level in the conceptualization of ORC as a further unit of analysis. Similarly, Weiner et al. (2020) argue for ORC being important to consider at the individual, group, and organizational level, while Vakola (2013) reports that groups influence organizational members’ behaviors. Our SLR adds further to this debate by highlighting that such an intermediate team level rarely is the focus of studies examining ORC and related factors. In our sample, little attention was paid to the role of teams in (a) ORC measurements and (b) the factors investigated in relation to ORC. As the unit responsible for or affected by an implementation, the team is only limitedly represented in five studies’ ORC measurements (e.g., Akande et al., 2019; Scales et al., 2017). Further, only one study (Rodriguez et al., 2016), as part of the factors reported alongside ORC, examined the relations between teamwork, team member availability, and ORC in an effort to improve diabetes care in a community health center. However, if, as suggested previously, implementation is understood as a team sport, involving different team members with distinct responsibilities, examining team characteristics, dynamics, and functioning in implementation, would contribute to the knowledge base surrounding ORC and provide an opportunity to enhance implementation science (Chan, 2019).

Finally, this SLR confirms ORC measurement challenges highlighted in previous publications (Gagnon et al., 2014; Weiner et al., 2008). While there is increasing consensus that ORC can fluctuate throughout implementation (Scaccia et al., 2015; Weiner et al., 2020), its measurement continues to be primarily based on single (baseline and/or retrospective) timepoints. These flaws—retrospective and single-time assessments—are inconsistent with how ORC is inherently understood, as reflected in common sense and the scientific understanding (Scaccia et al., 2015; Weiner, 2020).

In everyday language the term “readiness” suggests future orientation, making retrospective inquiry about “being ready” nonsensical. Consequently, this prevents meaningful conclusions about change implementation. Additionally, many ORC measures are self-report measures, and hence subject to social desirability and/or recall bias and may even be affected by respondents’ situational mood (Martinez et al., 2014; Podsakoff et al., 2003). This emphasizes a need to integrate and triangulate multiple data types, for example, observational and administrative data, when measuring ORC (Martinez et al., 2014).

Furthermore, implementation is a nonlinear process (Rapport et al., 2018), during which change is implemented sequentially, to reach extended uptake beyond the “logical endpoint of implementable interventions” (Rapport et al., 2018, p. 119; Figure 1). Accordingly, change is likely implemented in multiple stages, necessitating repeated ORC measurements at different timepoints, since ORC may differ as organizations and their members move through different stages of a change.

In summary, the synthesis of studies included in this SLR depicts the current state of ORC research, calling for improved conceptual clarity around ORC, more nuanced theorizing, and for considering the team level. This need for more rigorous ORC research is further mirrored in the generally only moderate assessment of the quality of included studies, pointing to limitations in especially quantitative studies, as well as quantitative and mixed-methods sections of mixed-methods studies.

### Implications

This SLR contributes to our understanding of ORC by identifying a collection of factors that have been investigated in relation to ORC in health care. As many constructs overlap with ORC (Bouckenooghe, 2010; Weiner, 2020), measuring ORC in isolation from other potentially relevant factors runs the risk of overlooking otherwise important influences on change implementation and thus of developing flawed explanations for implementation results. Although we found suboptimal study quality in line with prior critiques in the field, the factors emerging from this work provide a starting point for their selection and subsequent investigation in combination with ORC to provide richer interpretation and contribute to a deeper understanding of implementation outcomes.

In doing so, implementation scientists should build future ORC studies on transparent and coherent definitions of ORC, preferably using ORC theories to enhance the scientific knowledge base. Furthermore, empirical tests of ORC theories prospectively linking ORC to implementation outcomes are urgently needed to advance current best knowledge on whether and under which conditions ORC matters (Weiner, 2020). Ideally, this work would also focus more strongly on how to understand and measure ORC at the team level and give room to longitudinal research allowing to study ORC prospectively, over the course of multiple implementation stages, using multiple measurement points.

While waiting for this development of a more consolidated evidence base, implementation (support) practitioners are well advised in utilizing the scarce but nevertheless existing evidence to inform their work with considering, assessing and developing ORC prior to and during implementation processes. This implies using already existing ORC models, theories, and measures of greatest relevance to a given setting. In monitoring ORC and using ORC data to inform practice decisions, the use of these measures can be complemented with information gathered through previous experience with implementing change in an organization, observations, or other data collection. This will also allow for internal and external stakeholder engagement at different stages of change implementation, which is generally recommended (Gopichandran et al., 2016) and relevant to ensure continuity of implementation (Pellecchia et al., 2022). Stakeholder engagement may be of particular value when ORC remains fragile and requires to be reassessed, for example, when change implementation is disrupted due to unexpected local circumstances or failed implementation strategies.

### Limitations

This study has limitations that should be considered when using the presented findings. First, we may have missed factors investigated in relation to ORC or as possible moderators or mediators between ORC and implementation, as we (a) included studies from the healthcare sector only, and (b) excluded studies that measured ORC qualitatively. Second, we excluded studies that measured ORC only, without linking ORC to potential correlates or predictors, or without exploring potential mediators or moderators between ORC and implementation. However, as shown in Miake-Lye et al. (2020), some ORC assessments capture a broader range of constructs than others. ORC studies that were excluded because they did not investigate further factors, may have used broader ORC assessments that include potentially relevant factors, which we may have missed. This leads to a third limitation related to our framework use. We mapped identified SFs to TORC-CFIR framework components. However, considering the items of ORC assessment instruments used in included studies was out of scope of this SLR. Investigating these in greater detail would have helped to uncover potential overlap between ORC assessment items and SFs, thereby also identifying factors that are not part of any ORC assessment but may be relevant to investigate alongside ORC, further detailing the overview provided through this SLR. Taking these leads further into future ORC-focused SLR work would be a meaningful contribution.

## Conclusions

This systematic literature review of studies examining factors in relation to ORC highlights the still somewhat fuzzy boundaries that characterize ORC as it is reported and discussed in implementation science. Despite existing ORC theories, measures, and studies, subtle conceptual differences impact how ORC and related factors can be categorized, understood, and utilized to unify current best ORC knowledge. While we provide an overview of factors potentially relevant alongside ORC to interpreting implementation results, the precise role of ORC in implementation remains unclear. Our findings suggest that the assumption that ORC influences implementation needs reevaluation, calling for enhanced collaboration between implementation science and practice to enable ORC implementation research of greater conceptual clarity, rigor, and relevance.

## Supplemental Material

sj-pdf-1-irp-10.1177_26334895251334536 - Supplemental material for Organizational readiness for change: A systematic review of the healthcare literature Supplemental material, sj-pdf-1-irp-10.1177_26334895251334536 for Organizational readiness for change: A systematic review of the healthcare literature by Laura Caci, Emanuela Nyantakyi, Kathrin Blum, Ashlesha Sonpar, Marie-Therese Schultes, Bianca Albers and Lauren Clack in Implementation Research and Practice

sj-pdf-2-irp-10.1177_26334895251334536 - Supplemental material for Organizational readiness for change: A systematic review of the healthcare literature Supplemental material, sj-pdf-2-irp-10.1177_26334895251334536 for Organizational readiness for change: A systematic review of the healthcare literature by Laura Caci, Emanuela Nyantakyi, Kathrin Blum, Ashlesha Sonpar, Marie-Therese Schultes, Bianca Albers and Lauren Clack in Implementation Research and Practice

sj-pdf-3-irp-10.1177_26334895251334536 - Supplemental material for Organizational readiness for change: A systematic review of the healthcare literature Supplemental material, sj-pdf-3-irp-10.1177_26334895251334536 for Organizational readiness for change: A systematic review of the healthcare literature by Laura Caci, Emanuela Nyantakyi, Kathrin Blum, Ashlesha Sonpar, Marie-Therese Schultes, Bianca Albers and Lauren Clack in Implementation Research and Practice

sj-pdf-4-irp-10.1177_26334895251334536 - Supplemental material for Organizational readiness for change: A systematic review of the healthcare literature Supplemental material, sj-pdf-4-irp-10.1177_26334895251334536 for Organizational readiness for change: A systematic review of the healthcare literature by Laura Caci, Emanuela Nyantakyi, Kathrin Blum, Ashlesha Sonpar, Marie-Therese Schultes, Bianca Albers and Lauren Clack in Implementation Research and Practice

sj-pdf-5-irp-10.1177_26334895251334536 - Supplemental material for Organizational readiness for change: A systematic review of the healthcare literature Supplemental material, sj-pdf-5-irp-10.1177_26334895251334536 for Organizational readiness for change: A systematic review of the healthcare literature by Laura Caci, Emanuela Nyantakyi, Kathrin Blum, Ashlesha Sonpar, Marie-Therese Schultes, Bianca Albers and Lauren Clack in Implementation Research and Practice
