# Supplementary material for: Organizational readiness for change: A systematic review of the healthcare literature
Source: Implement Res Pract. 2025 May 15;6:26334895251334536. doi: 10.1177/26334895251334536 (PMC12084713; doi:10.1177/26334895251334536)
Supplement: sj-pdf-2-irp-10.1177_26334895251334536 - Supplemental material for Organizational readiness for change: A systematic review of the healthcare literature [file sj-pdf-2-irp-10.1177_26334895251334536.pdf]

Thursday, August 18, 2022 1:39:55 PM

#	Query	Limiters/Expanders	Last Run Via	Results
S3	S1 AND S2	Expanders - Apply equivalent subjects<br>Search modes - Find all my search terms	Interface - EBSCOhost Research Databases<br>Search Screen - Advanced Search<br>Database - MEDLINE	1,470
S2	(MH "Health Facilities+") OR TI (health OR hospital* OR healthcare OR "primary care" OR "medical care") OR AB (health OR hospital* OR healthcare OR "primary care" OR "medical care") OR SO (health OR hospital* OR healthcare OR care)	Expanders - Apply equivalent subjects<br>Search modes - Find all my search terms	Interface - EBSCOhost Research Databases<br>Search Screen - Advanced Search<br>Database - MEDLINE	4,856,274
S1	((MH "Organizational Innovation" OR TI ((organization* OR organisation* OR institution*) N6 (innovation* OR change OR intervention* OR transformation*)) OR AB ((organization* OR organisation* OR institution*) N6 (innovation* OR change OR intervention* OR transformation*))) AND (TI (readiness OR ready OR willing* OR preparedness) OR AB (readiness OR ready OR willing* OR preparedness)) OR ((TI ((organization* OR organisation* OR institution*) N6 (readiness OR ready OR willing* OR preparedness)) OR AB ((organization* OR organisation* OR institution*) N6 (readiness OR ready OR willing* OR preparedness))) AND (TI (innovation* OR change OR intervention* OR transformation*) OR AB	Expanders - Apply equivalent subjects<br>Search modes - Find all my search terms	Interface - EBSCOhost Research Databases<br>Search Screen - Advanced Search<br>Database - MEDLINE	1,830

(innovation* OR change OR
intervention* OR
transformation*))

Embase®

Embase Session Results (18 Aug 2022)

No.	Query	Results
#7	#1 AND #2 NOT [conference abstract]/lim NOT ([chinese]/lim OR [japanese]/lim OR [russian]/lim)	1052
#6	#1 AND #2 NOT [conference abstract]/lim AND ([chinese]/lim OR [japanese]/lim OR [russian]/lim)	3
#5	#1 AND #2 NOT [conference abstract]/lim	1055
#4	#1 AND #2 AND [conference abstract]/lim	276
#3	#1 AND #2	1331
#2	'health care facilities and services'/exp OR health:ti,ab,kw,jt OR hospital*:ti,ab,kw,jt OR healthcare:ti,ab,kw,jt OR 'primary care':ti,ab,kw,jt OR 'medical care':ti,ab,kw,jt	10524450
#1	(((organization* OR organisation* OR institution*) NEAR/6 (innovation* OR change OR intervention* OR transformation*)):ti,ab,kw) AND ('attitude to change'/exp OR readiness:ti,ab,kw OR ready:ti,ab,kw OR willing*:ti,ab,kw OR preparedness:ti,ab,kw) OR ((((organization* OR organisation* OR institution*) NEAR/6 (readiness OR ready OR willing* OR preparedness)):ti,ab,kw) AND (innovation*:ti,ab,kw OR change:ti,ab,kw OR intervention*:ti,ab,kw OR transformation*:ti,ab,kw))	1491

ELSEVIER

© 2022 Elsevier Life Sciences IP Limited except certain content provided by third parties.
Embase is a trade mark of Elsevier Life Sciences IP Limited. RELX Group and the RE symbol are trade marks of RELX Group plc, used under license.

EBSCOhost

Thursday, August 18, 2022 1:49:27 PM

#	Query	Limiters/Expanders	Last Run Via	Results
S3	S1 AND S2	Expanders - Apply equivalent subjects<br>Search modes - Find all my search terms	Interface - EBSCOhost Research Databases<br>Search Screen - Advanced Search<br>Database - CINAHL with Full Text	1,446
S2	(MH "Health Facilities+") OR TI (health OR hospital* OR healthcare OR "primary care" OR "medical care") OR AB (health OR hospital* OR healthcare OR "primary care" OR "medical care") OR SO (health OR hospital* OR healthcare OR care)	Expanders - Apply equivalent subjects<br>Search modes - Find all my search terms	Interface - EBSCOhost Research Databases<br>Search Screen - Advanced Search<br>Database - CINAHL with Full Text	2,603,208
S1	(((MH "Organizational Change") OR (MH "Organizational Development+") OR TI ((organization* OR organisation* OR institution*) N6 (innovation* OR change OR intervention* OR transformation*)) OR AB ((organization* OR organisation* OR institution*) N6 (innovation* OR change OR intervention* OR transformation*))) AND (MH "Attitude to Change" OR TI (readiness OR ready OR willing* OR preparedness) OR AB (readiness OR ready OR willing* OR preparedness)) OR ((TI ((organization* OR organisation* OR institution*) N6 (readiness OR ready OR willing* OR preparedness)) OR AB ((organization* OR organisation* OR institution*) N6 (readiness OR ready OR willing* OR preparedness)))	Expanders - Apply equivalent subjects<br>Search modes - Find all my search terms	Interface - EBSCOhost Research Databases<br>Search Screen - Advanced Search<br>Database - CINAHL with Full Text	2,127

AND (TI (innovation* OR change OR intervention* OR transformation*) OR AB (innovation* OR change OR intervention* OR transformation*))

Thursday, August 18, 2022 2:19:45 PM

#	Query	Limiters/Expanders	Last Run Via	Results
S3	(DE "Treatment Facilities" OR DE "Clinics" OR DE "Community Mental Health Centers" OR DE "Facility Admission" OR DE "Facility Discharge" OR DE "Halfway Houses" OR DE "Hospitals" OR DE "Nursing Homes" OR DE "Rehabilitation Centers" OR DE "Therapeutic Camps" OR DE "Child Guidance Clinics" OR DE "Psychiatric Clinics" OR DE "Walk In Clinics" OR DE "Psychiatric Hospitals" OR DE "Sanatoriums" OR DE "Nursing Home Residents" OR DE "Sheltered Workshops" OR TI (health OR hospital* OR healthcare OR "primary care" OR "medical care") OR AB (health OR hospital* OR healthcare OR "primary care" OR "medical care") OR SO (health OR hospital* OR healthcare OR care)) AND (S1 AND S2)	Expanders - Apply equivalent subjects<br>Search modes - Find all my search terms	Interface - EBSCOhost Research Databases<br>Search Screen - Advanced Search<br>Database - APA PsycInfo	547
S2	DE "Treatment Facilities" OR DE "Clinics" OR DE "Community Mental Health Centers" OR DE "Facility Admission" OR DE "Facility Discharge" OR DE "Halfway Houses" OR DE "Hospitals" OR DE "Nursing Homes" OR DE "Rehabilitation Centers" OR DE "Therapeutic Camps" OR DE "Child Guidance Clinics" OR DE "Psychiatric Clinics" OR DE "Walk In Clinics" OR DE	Expanders - Apply equivalent subjects<br>Search modes - Find all my search terms	Interface - EBSCOhost Research Databases<br>Search Screen - Advanced Search<br>Database - APA PsycInfo	952,276

	"Psychiatric Hospitals" OR DE "Sanatoriums" OR DE "Nursing Home Residents" OR DE "Sheltered Workshops" OR TI (health OR hospital* OR healthcare OR "primary care" OR "medical care") OR AB (health OR hospital* OR healthcare OR "primary care" OR "medical care") OR SO (health OR hospital* OR healthcare OR care)			
S1	((DE "Innovation" OR DE "Organizational Development" OR TI ((organization* OR organisation* OR institution*) N6 (innovation* OR change OR intervention* OR transformation*)) OR AB ((organization* OR organisation* OR institution*) N6 (innovation* OR change OR intervention* OR transformation*))) AND (DE "Employee Engagement" OR DE "Employee Motivation" OR TI (readiness OR ready OR willing* OR preparedness) OR AB (readiness OR ready OR willing* OR preparedness)) OR ((TI ((organization* OR organisation* OR institution*) N6 (readiness OR ready OR willing* OR preparedness)) OR AB ((organization* OR organisation* OR institution*) N6 (readiness OR ready OR willing* OR preparedness))) AND (TI (innovation* OR change OR intervention* OR transformation*) OR AB (innovation* OR change OR intervention* OR transformation*))	Expanders - Apply equivalent subjects<br>Search modes - Find all my search terms	Interface - EBSCOhost Research Databases<br>Search Screen - Advanced Search<br>Database - APA PsycInfo	2,265

Thursday, August 18, 2022 2:17:37 PM

#	Query	Limiters/Expanders	Last Run Via	Results
S3	S1 AND S2	Expanders - Apply equivalent subjects<br>Search modes - Find all my search terms	Interface - EBSCOhost Research Databases<br>Search Screen - Advanced Search<br>Database - Business Source Premier	296
S2	DE "Treatment Facilities" OR DE "Clinics" OR DE "Community Mental Health Centers" OR DE "Facility Admission" OR DE "Facility Discharge" OR DE "Halfway Houses" OR DE "Hospitals" OR DE "Nursing Homes" OR DE "Rehabilitation Centers" OR DE "Therapeutic Camps" OR DE "Child Guidance Clinics" OR DE "Psychiatric Clinics" OR DE "Walk In Clinics" OR DE "Psychiatric Hospitals" OR DE "Sanatoriums" OR DE "Nursing Home Residents" OR DE "Sheltered Workshops" OR TI (health OR hospital* OR healthcare OR "primary care" OR "medical care") OR AB (health OR hospital* OR healthcare OR "primary care" OR "medical care") OR SO (health OR hospital* OR healthcare OR care)	Expanders - Apply equivalent subjects<br>Search modes - Find all my search terms	Interface - EBSCOhost Research Databases<br>Search Screen - Advanced Search<br>Database - Business Source Premier	1,004,264
S1	((DE "ORGANIZATIONAL change" OR TI ((organization* OR organisation* OR institution*) N6 (innovation* OR change OR intervention* OR transformation*)) OR AB ((organization* OR organisation* OR institution*)	Expanders - Apply equivalent subjects<br>Search modes - Find all my search terms	Interface - EBSCOhost Research Databases<br>Search Screen - Advanced Search<br>Database - Business Source Premier	2,916

N6 (innovation* OR change OR intervention* OR transformation*))) AND (DE "EMPLOYEE attitudes" OR TI (readiness OR ready OR willing* OR preparedness) OR AB (readiness OR ready OR willing* OR preparedness)) OR ((TI ((organization* OR organisation* OR institution*) N6 (readiness OR ready OR willing* OR preparedness)) OR AB ((organization* OR organisation* OR institution*) N6 (readiness OR ready OR willing* OR preparedness))) AND (TI (innovation* OR change OR intervention* OR transformation*) OR AB (innovation* OR change OR intervention* OR transformation*))
