# Supplementary material for: Organizational readiness for change: A systematic review of the healthcare literature
Source: Implement Res Pract. 2025 May 15;6:26334895251334536. doi: 10.1177/26334895251334536 (PMC12084713; doi:10.1177/26334895251334536)
Supplement: sj-pdf-3-irp-10.1177_26334895251334536 - Supplemental material for Organizational readiness for change: A systematic review of the healthcare literature [file sj-pdf-3-irp-10.1177_26334895251334536.pdf]

Supplement C

Table C1. Quality criteria ratings for quantitative studies (n = 30).

**Quantitative descriptive studies** *First author, publication year*	Is the sampling strategy relevant to address the research question?	Is the sample representative of the target population?	Are the measurements appropriate?	Is the risk of nonresponse bias low?	Is the statistical analysis appropriate to answer the research question?
Abrahamsen, 2017	Y	Y	Y	Y	Y
Adelson, 2021	Y	Y	Y	C	Y
Akande, 2019	Y	C	Y	Y	Y
Alameddine, 2015	Y	Y	Y	N	Y
Birnie, 2022	Y	Y	Y	C	Y
Bohman, 2008	Y	C	Y	C	Y
Briggs, 2022	Y	N	N	N	Y
Cunha-Cruz, 2017	Y	N	Y	N	Y
Dönmez, 2020	C	C	Y	C	Y
Goebel, 2020	Y	C	Y	C	Y
Guerrero, 2020	Y	C	C	N	Y
Harrison, 2022	C	C	Y	C	Y
Hoffmann, 2022	Y	Y	Y	Y	Y
Kujala, 2019	Y	Y	Y	C	Y
Le, 2021	Y	C	Y	C	Y

**Randomized controlled trials** *First author, publication year*	Is randomization appropriately performed?	Are the groups comparable at baseline?	Are there complete outcome data?	Are outcome assessors blinded to the intervention provided?	Did the participants adhere to the assigned intervention?
Jakobsen, 2020	Y	Y	C	C	Y
von Treuer, 2022	C	Y	C	C	C

**Non-randomized studies** *First author, publication year*	Are the participants representative of the target population?	Are measurements appropriate regarding both the outcome and intervention (or exposure)?	Are there complete outcome data?	Are the confounders accounted for in the design and analysis?	During the study period, is the intervention administered (or exposure occurred) as intended?
Becker, 2016	Y	Y	C	Y	C

Myers, 2017	Y	Y	C	C	Y
Pinto, 2011	Y	Y	N	N	Y
Randall, 2020	Y	Y	Y	Y	Y
Rodriguez, 2016	Y	C	C	N	Y
Saleh, 2016	Y	Y	Y	Y	Y
Scales, 2017	C	Y	Y	C	Y
Smelson, 2022	Y	C	Y	C	Y
Spalluto, 2021	C	C	Y	C	Y
Stadnick, 2022	Y	C	Y	C	Y
Washington, 2018	C	C	Y	Y	Y
Williams, 2014	Y	Y	Y	Y	Y
Zullig, 2013	Y	Y	C	Y	Y

Y indicates a 'Yes' rating, C indicates a 'Can't tell' rating, N indicates a 'No' rating.

Supplement C

Table C2. Quality criteria ratings for mixed methods studies (n = 17).

Mixed methods studies *First author, publication year*	Qualitative elements					Quantitative descriptive elements					Mixed methods elements				
	Is the qualitative approach appropriate to answer the research question?	Are the qualitative data collection methods adequate to address the research question?	Are the findings adequately derived from the data?	Is the interpretation of results sufficiently substantiated by data?	Is there coherence between qualitative data sources, collection, analysis and interpretation?	Is the sampling strategy relevant to address the research question?	Is the sample representative of the target population?	Are the measurements appropriate?	Is the risk of nonresponse bias low?	Is the statistical analysis appropriate to answer the research question?	Is there an adequate rationale for using a mixed methods design to address the research question?	Are the different components of the study effectively integrated to answer the research question?	Are the outputs of the integration of qualitative and quantitative components adequately interpreted?	Are divergences and inconsistencies between quantitative and qualitative results adequately addressed?	Do the different components of the study adhere to the quality criteria of each tradition of the methods involved?
Burnett, 2010	Y	Y	Y	Y	Y	Y	C	Y	Y	Y	N	Y	Y	C	C
Chang, 2013	C	C	N	N	N	Y	Y	Y	Y	Y	Y	N	C	C	N
Chang, 2023	Y	Y	Y	Y	Y	C	Y	Y	Y	Y	Y	Y	Y	C	C
Elango, 2018	Y	Y	Y	Y	Y	Y	Y	Y	Y	Y	Y	Y	Y	Y	Y
Gallant, 2023	Y	Y	Y	Y	Y	Y	C	Y	C	Y	Y	N	Y	C	C
Garner, 2022	Y	Y	Y	Y	Y	Y	Y	Y	C	Y	Y	Y	Y	C	C
Geerligs, 2021	Y	Y	Y	Y	Y	Y	Y	Y	N	Y	Y	Y	Y	C	N
Hearld, 2022	Y	Y	Y	Y	Y	Y	Y	Y	Y	Y	Y	Y	Y	Y	Y
Joudrey, 2020	Y	Y	Y	Y	Y	Y	C	Y	Y	Y	N	Y	Y	Y	C
Lundgren, 2012	Y	Y	C	C	C	Y	Y	Y	Y	Y	N	Y	N	C	C
Lundgren, 2013	Y	Y	C	C	C	Y	Y	Y	Y	Y	N	Y	N	C	C
Messer, 2012	Y	Y	Y	Y	Y	Y	C	Y	Y	Y	N	C	Y	C	C
Peracca, 2021	Y	Y	Y	Y	Y	C	C	Y	C	Y	Y	C	C	C	C
Peracca, 2022	Y	Y	Y	Y	Y	C	C	Y	C	Y	N	Y	Y	C	C

Shrubsole, 2022	C	Y	Y	Y	Y	Y	C	Y	C	Y	Y	Y	Y	C	C
Stanhope, 2019	Y	Y	Y	Y	Y	C	C	N	C	Y	Y	Y	Y	C	N
Zapka, 2013	Y	Y	Y	C	Y	Y	Y	C	C	Y	Y	Y	Y	Y	C

Y indicates a 'Yes' rating, C indicates a 'Can't tell' rating, N indicates a 'No' rating.
