# Supplementary material for: Organizational readiness for change: A systematic review of the healthcare literature
Source: Implement Res Pract. 2025 May 15;6:26334895251334536. doi: 10.1177/26334895251334536 (PMC12084713; doi:10.1177/26334895251334536)
Supplement: sj-pdf-4-irp-10.1177_26334895251334536 - Supplemental material for Organizational readiness for change: A systematic review of the healthcare literature [file sj-pdf-4-irp-10.1177_26334895251334536.pdf]

Supplement D

Table D1. Overview of studies that contributed to synthesized factors.

Synthesized factors (SFs)	First author, publication year	Number of entries[1]	Number of studies
Acceptability	Briggs, 2021	1	1
Access to interdisciplinary expertise	Rodriguez, 2016	1	1
Change aligning with leadership goals	Peracca, 2022	1	1
Change fits with the facility's approach to patient care	Peracca, 2022	1	1
Change or intervention-specific topics*	Briggs, 2022<br>Cunha-Cruz, 2017<br>Geerligs, 2021<br>Guerrero, 2020	4	4
Change valence*	Akande, 2019<br>Peracca, 2021<br>Peracca, 2022	3	3
Commitment*	Harrison, 2022<br>Peracca, 2022	2	2
Communication*	Elango, 2018<br>Garner, 2022	2	2
Complexity of the change	Chang, 2013	1	1
Confidence that the facility can implement the change	Peracca, 2022	1	1
Effectiveness/Impact*	Burnett, 2010<br>Gallant, 2023<br>Le, 2021<br>Pinto, 2011	5	4
Engagement*	Garner, 2022<br>Geerligs, 2021	2	2
Flexibility to change behaviors	Geerligs, 2021	1	1
Implementation climate*	Hoffmann, 2022<br>Stadnick, 2022	2	2
Implementation leadership	Stanhope, 2019	1	1
Implementation phase	Chang, 2023	1	1
Implementation preparation	Geerligs, 2021	1	1
Individual attitudes*	Alameddine, 2015<br>Guerrero, 2020<br>Peracca, 2022	4	3
Individual demographics*	Abrahamsen, 2017<br>Alameddine, 2015<br>Briggs, 2022<br>Dönmez, 2020<br>Hoffmann, 2022<br>Myers, 2017<br>Rodriguez, 2016<br>Saleh, 2016<br>Smelson, 2022<br>Washington, 2018	20	10
Individual readiness for change	Harrison, 2022	1	1
Job-related demographics*	Abrahamsen, 2017<br>Alameddine, 2015<br>Briggs, 2022<br>Hearld, 2022<br>Myers, 2017<br>Saleh, 2016<br>Smelson, 2022<br>Washington, 2018	15	8
Job-related stress*	Cunha-Cruz, 2017<br>Peracca, 2022	3	2

Job satisfaction*	Cunha-Cruz, 2017<br>Randall, 2020	2	2
Knowledge	Akande, 2019	1	1
Leadership attendance	Stanhope, 2019	1	1
Leadership support*	Kujala, 2019<br>Peracca, 2021<br>Peracca, 2022	3	3
Leaders vs. non-leaders	Spalluto, 2021	1	1
Level of barriers	Lundgren, 2012	1	1
Level of modifications made to the change	Lundgren, 2013	1	1
Likelihood to leave workplace	Cunha-Cruz, 2017	1	1
Medical specialty*	Abrahamsen, 2017<br>Hearld, 2022<br>Messer, 2012<br>Spalluto, 2021	4	4
Need of more provider buy-in	Peracca, 2022	1	1
Organizational climate*	Cunha-Cruz, 2017	2	1
Organizational context and resources	Randall, 2020	1	1
Organizational culture*	Geerligs, 2021<br>Hearld, 2022<br>Zapka, 2013	4	3
Organizational demographics*	Alameddine, 2015<br>Birnie, 2022<br>Bohman, 2008<br>Briggs, 2022<br>Dönmez, 2020<br>Hearld, 2022<br>Williams, 2014	11	7
Organizational functioning (emphasis on quality and management)	Randall, 2020	1	1
Organizational implementation challenges	Garner, 2022	1	1
Patient-related topics*	Peracca, 2022	2	1
Pre-existing shared practice behavior	Elango, 2018	1	1
Presence of a clinical champion	Peracca, 2022	1	1
Proactive versus passive effort to improve	Elango, 2018	1	1
Professional role*	Abrahamsen, 2017<br>Adelson, 2021<br>Alameddine, 2015<br>Bohman, 2008<br>Cunha-Cruz, 2017<br>Dönmez, 2020<br>Goebel, 2020<br>Guerrero, 2020<br>Hoffmann, 2022<br>Joudrey, 2020<br>Messer, 2012<br>Myers, 2017<br>Randall, 2020<br>Saleh, 2016<br>Scales, 2017<br>Smelson, 2022<br>Spalluto, 2021<br>Washington, 2018<br>Williams, 2014<br>Zullig, 2013	24	20
Resource Availability*	Akande, 2019<br>Peracca, 2021<br>Peracca, 2022<br>Saleh, 2016<br>Zapka, 2013	5	5
Self-efficacy*	Guerrero, 2020	2	2

	Harrison, 2022		
Situational factors*	Gallant, 2023<br>Peracca, 2021<br>Peracca, 2022	3	3
Social influence	Gallant, 2023	1	1
Strong program already existing	Peracca, 2022	1	1
Support for the company's mission	Cunha-Cruz, 2017	1	1
Support for the change*	Cunha-Cruz, 2017<br>Guerrero, 2020<br>Peracca, 2022	3	3
Sustainability*	Burnett, 2010<br>Geerligs, 2021	2	2
Task demands*	Gallant, 2023<br>Peracca, 2022<br>Zapka, 2013	3	3
Team member availability	Rodriguez, 2016	1	1
Team size	Rodriguez, 2016	1	1
Teamwork	Rodriguez, 2016	1	1
Technology-related topics*	Dönmez, 2020<br>Hoffmann, 2022<br>Peracca, 2022<br>Saleh, 2016	5	4
Top-down vs. bottom-up approach to implementation	Burnett, 2010	1	1
Use of certain implementation strategies*	Becker, 2016<br>Jakobsen, 2020<br>Shrubsole, 2022<br>Von Treuer, 2022	4	4

** Indicates that the synthesized factor was composed of multiple raw factors coming from multiple studies (i.e., according to synthesis step 1 in methodology section of main article); [1]The number of entries indicates the number of times a synthesized factor was represented across included studies.*
