# Supplementary material for: Organizational readiness for change: A systematic review of the healthcare literature
Source: Implement Res Pract. 2025 May 15;6:26334895251334536. doi: 10.1177/26334895251334536 (PMC12084713; doi:10.1177/26334895251334536)
Supplement: sj-pdf-5-irp-10.1177_26334895251334536 - Supplemental material for Organizational readiness for change: A systematic review of the healthcare literature [file sj-pdf-5-irp-10.1177_26334895251334536.pdf]

Supplement E

Table E1. Overview of ORC sample and team-level considerations in ORC measurements.

First author, publication year	ORC measure (subscales)	Description of ORC sample	Considerations of the team-level in ORC measurement
Abrahamsen, 2017	Adaptation from Kristensen and Nøhr (2000) (knowledge and understanding, need for change, readiness for change, planning for change)	*"All health care professionals who were considered to have a professional relationship with orthogeriatric patients in terms of treatment or administration were included. They were physicians, nurses, nursing assistants, physiotherapists, occupational therapists, secretaries, dieticians and managers from the departments of medicine, orthopaedic surgery and therapy."* (Abrahamsen et al., 2017, p. 19)	None reported
Adelson, 2021	ORIC [1] (change commitment, change efficacy)	*"The survey was distributed anonymously in August 2019 to 102 clinicians working directly in the MoC [Model of Care] or those impacted by the changes; midwives (n = 12) and doctors (n = 10) transitioning to the model of care, and to midwives and nurses providing direct maternity care at the 5 local hospitals (n = 80)."* (Adelson et al., 2021, p. 3)	None reported
Akande, 2019	Adapted ORIC[1] (subscales not reported)	*"The ORIC questionnaire was distributed to all public/ population health and health policy personnel who were responsible for policy or program development, implementation, and evaluation in the Department of Health."* (Akande et al., 2019, p. 3)	Group-referencing of items, such as 'we know…' as opposed to 'I know…', to draw respondents' attention to *"the team's collective readiness rather than personal readiness of individuals."* (Akande et al., 2019, p. 2)
Alameddine, 2015	Readiness for Organization Change (ROC) scale[2] (appropriateness of performance reporting, management support, efficacy, personal valence)	*"[…] a third of participating primary health-care providers were specialists (33 %), followed by nurses (25 %), allied health professionals (18 %) and dentists and family physicians (12 % each)."* (Alameddine et al., 2015, p. 6)	None reported
Becker, 2016	ORC-S[3] (motivation for change, adequacy of resources, staff attributes, organizational climate)	*"The final sample contained 60 providers across 15 sites (7 SSL [Science to Service Laboratory], 8 TAU [Training As Usual])."* (Becker et al., 2016, p. 4)	None reported

Birnie, 2022	ORIC[1] (change commitment, change efficacy)	*"The remaining 85 [respondents] (72.0%) worked at a health care institution that conducted pediatric surgery. Reported results are based on this sample of 85 participants."* (Birnie et al., 2022, p. 112)	None reported
Bohman, 2008	MORC[4], adapted from ORC-S[3] (need for external guidance, pressure to change, organizational readiness to change, individual readiness to change, workgroup functioning, work environment, autonomy support, and alcohol and drug focus)	*Evaluators administered MORC surveys at 4 SBIRT implementation sites (3 CHP clinics and 1 EC) according to recommendations arising from the stakeholder analysis process. For example, 1 CHP chose to ask those staff most likely to be directly involved in project implementation to complete the MORC, whereas the 2 other CHPs chose to administer the MORC to a wider staff audience. Medical and ancillary staff completed the surveys anonymously."* (Bohman et al., 2008, p. 152)	ORC level was further considered and defined based on a stakeholder analysis process, but to which degree ORC measurements represented the team level was not reported
Briggs, 2022	Adapted ORCA[5] (culture and resources)	*"We surveyed members of the Association of Professors of Dermatology […]."* (Briggs et al., 2022, p. 2)	None reported
Burnett, 2010	Respondents were asked 'How ready was your organization at the start of Safer Patients' Initiative (SPI) for successful implementation of the program?' (culture and attitudes towards quality and safety, systems and infrastructure, availability of resources)	*"A systematic sampling strategy was employed to include the SPI [improvement team in each organization comprising: the senior executive leads, the principal SPI programme coordinator and the operational leads in each of the clinical work areas. Individuals were recruited by local site coordinators."* (Burnett et al., 2010, p. 314)	The sample consisted of the SPI improvement team in each site. However, in the discussion section, it becomes clear that these teams rated ORC at the organizational level rather than at the team level: *"The teams leading the implementation of SPI perceived their organisations to be well prepared at the start in possessing the 'right' culture, attitudes and values, as well as having suitably motivated people to lead the changes."* (Burnett et al., 2010, p. 316)
Chang, 2013	ORC[3] (adequacy of resources, motivation for change, staff	*"We used data from the 2007 VA Clinical Practice Organization Survey (CPOS) Primary Care Directors Module, a nationwide survey of PC*	Clearly state organizational level in

	attributes, organizational climate)	*[Primary Care] directors on organization-level characteristics for their respective practices [Yano et al., 2008] to assess PC practice readiness."* (Chang et al., 2013, p. 354)	focus as compared to group or individual level.
Chang, 2023	ORIC[1] (change commitment, change efficacy)	*"[…] we administered the Organizational Readiness for Implementing Change (ORIC) to all team leaders."* (Chang et al., 2023, p. 3)	None reported
Cunha-Cruz, 2017	ORIC[1] (change commitment, change efficacy)	*"The sample frame was all providers and staff in the group and affiliated private dental practices in the 14 counties and administrative staff at the company's headquarters."* (Cunha-Cruz et al., 2017, p. 4)	None reported
Dönmez, 2020	OITIRS[6] (organizational readiness, technological readiness)	*"In this cross-sectional study, 236 medical employees […] and 139 administrative employees […] from 15 public health institutions in Kocaeli, Marmara Region were included."* (Dönmez et al., 2020, p. 266)	None reported
Elango, 2018	ORCA[5] (evidence, context, facilitation)	*"First, a survey was administered to assess a practice's readiness to change for the intervention."* (Elango et al., 2017, p. 155)<br>*[…] "The survey instrument was administered electronically to all clinicians […]."* (Elango et al., 2017, p. 155)	None reported
Gallant, 2023	ROC[2] (appropriateness, personal valence, management support, change efficacy)	*"Recruitment of participants resulted in an initial sample of 164 LTC [long-term care] nurses and a final sample of 157 LTC nurses."* (Gallant et al., 2023, p. 3)	None reported
Garner, 2022	ORIC[1] (change commitment, change efficacy)	*"A range of staff across the 4 clinics participated in each round of surveys and interviews."* (Garner et al., 2022, p. 50)	None reported
Geerligs, 2021	ORIC[1] (change commitment, change efficacy)	*"All staff were invited to complete the quantitative online survey […]."* (Geerligs et al., 2021, p. 3236)	None reported
Goebel, 2020	Adapted ORCA[5] (evidence, context)	*"Surveys were distributed to prescribing providers (residents, staff physicians, physician assistants, and nurse practitioners), nurses (registered nurses, licensed vocational nurses, and licensed practical nurses), clinical nurse assistants (CNA), infection preventionists, and quality managers."* (Goebel et al., 2020, p. 4)	None reported
Guerrero, 2020	Attitude toward change instrument[7] (subscales not reported)	*"Our sampling frame included 430 ED [emergency department] practitioners (physicians, nurses, and social workers) […]."* (Guerrero et al., 2020, p. 5)	None reported
Harrison, 2022	Self-developed, validated elements of a survey (subscales not reported)	*"Employees in any clinical or nonclinical role who were directly affected by or involved in the change/s proposed in each project were eligible to participate […]."* (Harrison et al., 2022, p. 604)	None reported
Hearld, 2022	ORIC[1] (change commitment, change efficacy)	*"[…] a web-based survey of members of the clinics, including physicians, nurses, clinic administrators, medical assistants, front desk personnel and research study coordinators."* (Hearld et al., 2022, p. 124)	None reported
Hoffmann, 2022	Organizational Change Questionnaire[8] (Readiness for	*"[…] a survey with a lead nurse and a lead physician from every NICU [neonatal intensive care unit] in Germany."* (Hoffmann et al., 2022, p. 3)	None reported

	Change - emotional and intentional readiness)		
Jakobsen, 2020	ORIC[1] (change commitment, change efficacy)	*"Finally, a total of 27 departments with 625 healthcare workers were willing to participate."* (Jakobsen, p. 2687-2688)	None reported
Joudrey, 2020	Adapted ORCA[5] (evidence, context)	*"We purposively sampled non-prescribing (registered nurse, pharmacist, and social work) and prescribing (physician or advanced practice provider hospitalist, general internist, and psychiatrist) professionals […] on the general medicine inpatient service."* (Joudrey et al., 2020, p. 3)	None reported
Kujala, 2019	Organizational readiness scale[9] (subscales not reported)	*"Responses from 44 organizational units (eg, primary care in Helsinki) were included in the analysis. All together, 401 leader and 2067 health professional respondents working in these organizational units responded to the questionnaire."* (Kujala et al., 2019, p. 4)	None reported
Le, 2021	Adapted ORIC[1] (resource availability, change efficacy, change valence, change commitment, task knowledge)	15 Substance Use Treatment Centers, with 525 respondents to questionnaires (clinicians and general employees with no patient contact)	None reported
Lundgren, 2012	TCU ORC[10] - staff and director versions (motivation for change, adequacy of resources, staff attributes, and organizational climate)	*"Our two samples included 212 program directors and 312 staff […]."* (Lundgren et al., 2012, p. 273)	None reported
Lundgren, 2013	TCU ORC[10]- staff (motivation for change, adequacy of resources, staff attributes, and organizational climate)	*"[…] Web surveys were conducted with a sample of 524 clinical staff […]."* (Lundgren et al., 2013, p. 459)	None reported
Messer, 2012	ORC[3] (ORC-SA and ORC-S, [motivation for change, adequacy of resources, staff attributes, and organizational climate])	"*Three different ORC forms were administered to directors and staff prior to CHIC RHIO [Carolina HIV Information Cooperative regional health information organization] development (June 2008).*" (Messer et al., 2012, p. e47)	None reported
Myers, 2017	Survey about readiness to adopt SQM initiative[11] (subscales not reported) and TCU ORC[10] (pressure to change)	*"The final sample comprised 81 participants with a diverse range of roles, including programme directors (n=7), clinical supervisors (n=3), counsellors (n=46), and support staff who conduct clinical intakes (n=26)."* (Myers et al., 2017, p. 161)	None reported
Peracca, 2021	ORIC[1] (change commitment, change efficacy)	*"The 12-question online survey assessed perceptions of organizational-level change efficacy and commitment to the app in end-users and other key implementation participants."* (Peracca et al., 2021, p. 1418)	None reported

Peracca, 2022	ORIC[1] (change commitment, change efficacy)	*"In total, 18 staff members from the 3 facilities participated to various extents in interviews and the ORIC survey."* (Peracca et al., 2022, p. 3)	None reported
Pinto, 2011	Self-developed ('How ready do you think your organization was for the SPI before it started?', followed by the presentation of eight items[12])	*"[…] local programme coordinators from the 20 sites were asked to compile a sample of relevant staff in their organizations based on criteria defined by the researchers. A systematic sampling strategy was employed to include the core SPI improvement team in each organization comprising: the senior executive leads, the SPI coordinators, operational leads in each clinical area and other staff involved within the programme."* (Pinto et al., 2011, p.175)	None reported
Randall, 2020	ORIC[1] (change commitment, change efficacy)	*"The sample pool included the dental director, dentists, dental therapists, dental health aides, dental hygienists, dental assistants, sterilization technicians, and administrative/management staff from the dental department."* (Randall et al., 2020, p. 158)	None reported
Rodriguez, 2016	Organizational Readiness for Change Assessment instrument[13] (change culture)	*"We conducted a survey of a census of adult primary care physicians, advanced practice clinicians, and staff with direct patient care responsibilities (n = 766 outgoing) in 34 community health centers (CHCs) […]."* (Rodriguez et al., 2016, p. 288)	None reported
Saleh, 2016	ROC[2] (appropriateness, management support, change efficacy, personally beneficial)	*"All primary healthcare providers practicing at the selected centers were eligible for inclusion in the study (n = 541), irrespective of their occupation, employment status, and length of time employed at the specific PHC [primary healthcare]. These included physicians, nurses, technicians, pharmacists, and nutritionists."* (Saleh et al., 2016, p. 4)	None reported
Scales, 2017	Adapted ORIC[1] (change commitment, change efficacy)	*"One hundred eighty-two nurses and 50 medical providers responded to the questionnaire […]."* (Scales et al., 2017, p. 167)	Results section may indicate that there were intentions to specifically measure ORC at the team level, i.e., *"Also, nurses rated the overall readiness of the nursing team more highly […]."* (Scales et al., 2017, p.168)
Shrubsole, 2022	ORIC[1] (change commitment, change efficacy)	*"A total of 35 SLTs [speech-language therapists] participated […]."* (Shrubsole et al., 2022, p. 160)	Acknowledge that ORIC represents measure of a group's ORC.
Smelson, 2022	Adapted ORCA[5] (context)	*"[…] 77 staff (69% Site A, 87% Site B) completed an organizational readiness and demographic survey. Most respondents*	None reported. Clearly state that *"data were measured at the site,*

		were case managers (77%) or peer specialists (16%) and few were unknown (8%).” (Smelson et al., 2022, p. 7)	*staff, and/or veteran level”* (Smelson et al., 2022, p. 6).
Spalluto, 2021	ORIC[1] (change commitment, change efficacy)	*“In this pilot study, we evaluated organizational readiness for change and change valence among clinical providers, staff, and administrators affiliated with radiology and primary care at a single VAMC [Veterans Affairs Medical Center].”* (Spalluto et al., 2021, p. 3)	None reported
Stadnick, 2022	Adapted ORIC[1] (change commitment, change efficacy)	*“Participants included 36 primary care providers (PCPs) from three healthcare organizations […].”* (Stadnick et al., 2022, p. 2)	None reported
Stanhope, 2019	Self-developed (single-item ten-point Likert scale [1 = low; 10 = high] in which consultants made a global assessment of a clinic’s readiness to implement the person-centered care planning intervention	*“Within these clinics, leadership, supervisors, and direct care staff participated in the study. These study participants came from various disciplines including social work, psychology and counselling.”* (Stanhope et al., 2019, p. 680)	None reported
Von Treuer, 2022	ROC[2] (appropriateness of performance reporting, management support, efficacy, personal valence)	*“Participants were 129 staff (M = 43.76 years; SD = 12.11 years; 110 women and 19 men) employed across 16 residential aged care facilities in Australia.”* (Von Treuer et al., 2022, p. 56)	None reported in their own ORC measurements, but when introducing ORC concept, Von Treuer et al. (2022) state that: *“In addition to individual-level change, change also needs to occur at a team-based or organizational level.”* (Von Treuer et al., 2022, p. 55)
Washington, 2018	ORIC[1] (change commitment, change efficacy) + one global rating question (‘How ready is your facility to implement this program?’)	*“Data for this study were collected from staff within three independently-managed outpatient dialysis facilities of an academic medical center in a major metropolitan area in the southeastern United States.”* (Washington et al., 2018, p. 3)	None reported
Williams, 2014	TCU ORC - staff and director versions[10] (motivation for change, program resources, staff attributes, organizational climate) and adapted	*“Three hundred eleven participants representing 92 organizations (49 community health, 43 community behavioral health) provided consent and completed baseline surveys, thus representing the final sample for the study.”* (Williams et al., 2014, p. 279)	None reported

	Organizational Readiness and Capacity assessment[14] (clients, leadership/clinicians/staff, supervision, internal and external stakeholders, program/culture/services, finance and administration, education, and technology)		
Zapka, 2013	Self-developed ('ED staff is receptive to CREST')	*"Respondents included physicians, nurses, EMS [unclear] personnel, and a sampling of other disciplines."* (Zapka et al., 2013, p. 4)	None reported
Zullig, 2013	Unclear, but subscales mentioned are change commitment, change efficacy, determinants of change efficacy	Respondents were physicians, nurses, residents, administrators, and medical students from different medical departments: Internal medicine, surgery, pediatrics, urology, medical records, gynecology, dermatology, pathology. (Zullig et al., 2013, Table 1)	None reported

[1] ORIC = Organizational Readiness for Implementing Change Scale (Shea et al., 2014); [2] ROC = Readiness for Organization Change (Holt et al., 2007a);[3] ORC = Organizational Readiness For Change Scale, the ORC-SA was designed for social service agencies and the ORC-S was designed for substance abuse treatment agencies (Lehman et al., 2002); [4] MORC = The Medical Organizational Readiness For Change (Bohman et al., 2008); [5] ORCA = Organizational readiness to change assessment instrument (Helfrich et al., 2009); [6] OITIRS = Organizational Information Technology Innovation Readiness Scale (Snyder-Halpern, 2002), of which only organizational readiness scale is considered for this SR; [7] Gardner et al. (1989); [8] Bouckenooghe et al. (2009); [9] Paré et al. (2011); [10] TCU ORC = Texas Christian University Organizational Readiness for Change scales (Lehman et al., 2002) ; [11] Items not reported, Myers et al. (2017); [12] Scale items of the self-developed measure included 'recognition of existing safety problems', 'knowledge of how to tackle safety problems', 'systems and infrastructure to support safety improvement' (Pinto et al., 2011); [13] source of instrument not reported. [14] Allred et al. (2005). Lundgren et al. (2013) builds on the previously conducted study by Lundgren et al. (2012). Peracca et al. (2021) and Peracca et al. (2023) are both studies as part of a larger trial (Done et al., 2018). Quotes for the description of the ORC sample were usually derived from the study's methods or results sections.
